# Caspase-Mediated Cleavage of the Transcription Factor Sp3: Possible Relevance to Cancer and the Lytic Cycle of Kaposi’s Sarcoma-Associated Herpesvirus

**DOI:** 10.1128/spectrum.01464-21

**Published:** 2022-01-12

**Authors:** Li-Yu Chen, Lee-Wen Chen, Chien-Hui Hung, Chun-Liang Lin, Shie-Shan Wang, Pey-Jium Chang

**Affiliations:** a Graduate Institute of Clinical Medical Sciences, College of Medicine, Chang-Gung University, Taoyuan, Taiwan; b Department of Respiratory Care, Chang-Gung University of Science and Technology, Chiayi, Taiwan; c Department of Pediatric Surgery, Chang-Gung Memorial Hospital, Chiayi, Taiwan; d Department of Nephrology, Chang-Gung Memorial Hospital, Chiayi, Taiwan; e School of Medicine, Chang-Gung University, Taoyuan, Taiwan; Barnard College, Columbia University

**Keywords:** KSHV, Sp3, ORF50, caspases, proteolytic cleavage, synergistic activation

## Abstract

The open reading frame 50 (ORF50) protein of Kaposi’s sarcoma-associated herpesvirus (KSHV) is the master regulator essential for initiating the viral lytic cycle. Previously, we have demonstrated that the ORF50 protein can cooperate with Sp3 to synergistically activate a set of viral and cellular gene promoters through highly conserved ORF50-responsive elements that harbor a Sp3-binding motif. Herein, we show that Sp3 undergoes proteolytic cleavage during the viral lytic cycle, and the cleavage of Sp3 is dependent on caspase activation. Since similar cleavage patterns of Sp3 could be detected in both KSHV-positive and KSHV-negative lymphoma cells undergoing apoptosis, the proteolytic cleavage of Sp3 could be a common event during apoptosis. Mutational analysis identifies 12 caspase cleavage sites in Sp3, which are situated at the aspartate (D) positions D17, D19, D180, D273, D275, D293, D304 (or D307), D326, D344, D530, D543, and D565. Importantly, we noticed that three stable Sp3 C-terminal fragments generated through cleavage at D530, D543, or D565 encompass an intact DNA-binding domain. Like the full-length Sp3, the C-terminal fragments of Sp3 could still retain the ability to cooperate with ORF50 protein to activate specific viral and cellular gene promoters synergistically. Collectively, our findings suggest that despite the proteolytic cleavage of Sp3 under apoptotic conditions, the resultant Sp3 fragments may retain biological activities important for the viral lytic cycle or for cellular apoptosis.

**IMPORTANCE** The ORF50 protein of Kaposi’s sarcoma-associated herpesvirus (KSHV) is the key viral protein that controls the switch from latency to lytic reactivation. It is a potent transactivator that can activate target gene promoters via interacting with other cellular DNA-binding transcription factors, such as Sp3. In this report, we show that Sp3 is proteolytically cleaved during the viral lytic cycle, and up to 12 caspase cleavage sites are identified in Sp3. Despite the proteolytic cleavage of Sp3, several resulting C-terminal fragments that have intact zinc-finger DNA-binding domains still retain substantial influence in the synergy with ORF50 to activate specific gene promoters. Overall, our studies elucidate the caspase-mediated cleavage of Sp3 and uncover how ORF50 utilizes the cleavage fragments of Sp3 to transactivate specific viral and cellular gene promoters.

## INTRODUCTION

Kaposi’s sarcoma-associated herpesvirus (KSHV) is a double-stranded DNA virus belonging to the gammaherpesvirus subfamily (1). This virus is closely associated with Kaposi’s sarcoma (KS), primary effusion lymphoma (PEL), and multicentric Castleman’s disease (2–4). Like other herpesviruses, KSHV exhibits two distinct life cycles, namely, latency and the lytic cycle (5, 6). Although the physiological stimuli that trigger KSHV lytic reactivation *in vivo* are not yet fully understood, various chemical agents or biological conditions, including sodium butyrate (SB), 12-*O*-tetradecanoylphorbol-13-acetate (TPA), high levels of glucose, proinflammatory cytokines, reactive oxygen species, hypoxia, or endoplasmic reticulum stress, have been reported to moderately induce viral reactivation in established PEL cell lines (5, 7–12). The switch of KSHV from latency to lytic replication is initiated by the expression of a transactivator encoded by the viral open reading frame 50 (ORF50) (13–15). The ORF50 protein, also known as RTA (replication and transcription activator), is the key regulator sufficient for driving the lytic cascade to completion (13–15). Numerous studies have shown that the ORF50 protein activates its downstream target genes through at least two distinct mechanisms (6, 15, 16). Activation of one subclass of viral target genes including PAN, K12, and oriLyt-T by ORF50 is mediated through a direct DNA-binding mechanism (17, 18). The other mechanism is mediated through indirect binding of ORF50 to target DNA elements via interacting with other cellular DNA-binding factors, such as RBP-Jκ, C/EBPα, Oct-1, or Stat3 (16, 19–22). Recently, our studies also revealed that ORF50 can cooperate with Sp3 to synergistically activate a subset of viral and cellular gene promoters (23). This transactivation mechanism utilized by ORF50 has been demonstrated for the viral ORF56 (primase), ORF21 (thymidine kinase), and ORF60 (ribonucleotide reductase, small subunit) promoters, as well as the cellular interleukin-10 (IL-10) promoter. Notably, all of the ORF50-responsive elements identified in these viral and cellular promoters harbor both a unique Sp3-binding motif (5′ACAAGGAGGGGCT) and a conserved flanking GT-rich motif (23).

Sp3, a member of the specificity protein/Kruppel-like factor (Sp/KLF) family, is expressed ubiquitously in all mammalian cells (24). Accumulating evidence has shown that Sp3 plays critical roles in various cellular processes, including cell growth, differentiation, survival, and migration (24–26). Like Sp1 and Sp4, Sp3 contains two glutamine-rich activation domains (also known as domains A and B) located at the N terminus and a highly conserved Cys2-His2 zinc-finger DNA-binding domain located at the C terminus (24). In addition to these functional domains, Sp3 has a unique transactivation inhibitory domain located between the second activation domain (domain B) and the DNA-binding domain (27). There are four major isoforms of Sp3 normally expressed in cells, including the longest isoform comprising 781 amino acid (aa) residues, and three shorter isoforms arising from alternative translation start codons at positions 13, 286, and 303 (28). Similar to Sp1 and Sp4, Sp3 is often highly expressed in cancer cells, and knockdown of Sp3 in these cancer cells dramatically reduced cell proliferation and survival (26, 29). However, Essafi-Benkhadir et al. (30) reported that overexpression of Sp3 in a colon carcinoma cell line LS174 significantly induced cell apoptosis, implicating that Sp3 may have a proapoptotic activity. Interestingly, following Sp3 overexpression in LS174 cells, they found that several cleavage products of Sp3 could be detected in apoptotic cells (30), thereby suggesting that Sp3 is a substrate of caspases. Although Sp3 could be a substrate of caspases, the precise positions of the caspase cleavage sites in Sp3 are completely unknown. Moreover, it is also unclear whether the resultant fragments of Sp3 have any intrinsic biological activities in cells.

Since Sp3 is involved in a wide variety of cellular processes and is required for ORF50-mediated transactivation, we aimed to further characterize the role of Sp3 in the viral lytic cycle. In this study, we report that Sp3 is proteolytically cleaved in PEL cells after KSHV reactivation, and the proteolytic cleavage of Sp3 is dependent on caspase activation. Furthermore, up to 12 caspase cleavage sites are found in Sp3. Upon proteolytic cleavage of Sp3 by caspases, we noticed that several cleaved C-terminal fragments of Sp3 were quite stable in cells and retained intact zinc-finger DNA-binding domain. Importantly, these C-terminal cleavage fragments of Sp3 were still able to cooperate with ORF50 protein to synergistically activate specific gene promoters. The possible relevance of the caspase-mediated cleavage of Sp3 to the progression of the viral lytic cycle is discussed in the study.

## RESULTS

### Proteolytic processing of Sp3 occurs during the KSHV lytic cycle.

**FIG 1:**
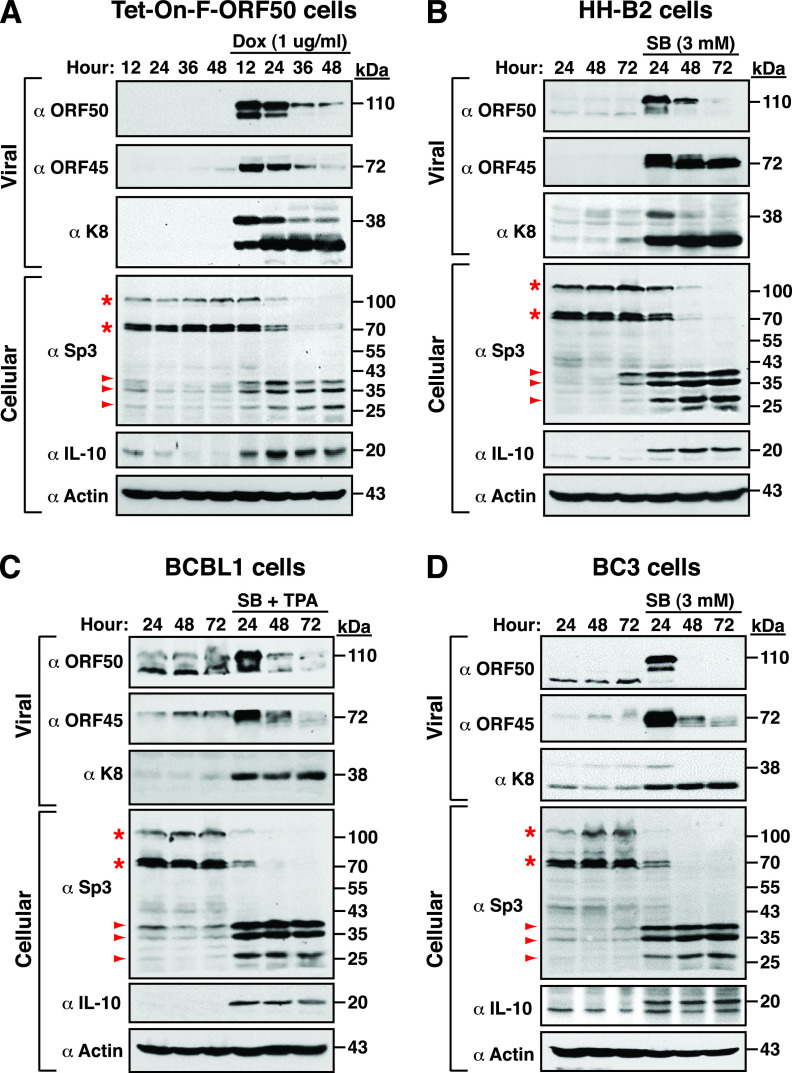
Sp3 undergoes proteolytic cleavage during the KSHV lytic cycle in PEL cells. Different PEL cell lines including Tet-On-F-ORF50 (A), HH-B2 (B), BCBL-1 (C), and BC3 (D) were left untreated or treated with doxycycline, SB, and the combination of SB and TPA, respectively, to induce the viral lytic cycle. At different time points after treatment, cells were harvested and analyzed for the expression of specific viral lytic proteins, including ORF50, ORF45, and K8, as well as cellular proteins, including Sp3 and IL-10 by Western blotting. Asterisks indicate the long and short isoforms of Sp3, and arrowheads indicate three major Sp3 cleavage products with the molecular masses of 38, 36, and 28 kDa.

To understand the expression kinetics of Sp3 during the viral lytic cycle, different PEL cell lines including Tet-On-F-ORF50, HH-B2, BCBL1, and BC3 were left untreated or treated with doxycycline, SB alone, and the combination of SB and TPA, respectively, to induce viral reactivation. As expected, the lytic gene products, such as ORF50, ORF45, and K8, could be abundantly expressed in these KSHV-positive cell lines after treatment with chemical inducers (Fig. 1A to D). Under the conditions, upregulation of IL-10 was also concomitantly detected in these treated cells (Fig. 1A to D). Unexpectedly, we found that levels of Sp3 (115 kDa) and its short isoforms (72 and 70 kDa) were significantly reduced in these chemical-treated cells, which were accompanied by increased levels of several small Sp3 fragments (Fig. 1A to D). Typically, there were three major Sp3 fragments with the estimated molecular masses of 38, 36, and 28 kDa detected in these chemical-treated cells. These results implicated that Sp3 might undergo proteolytic cleavage during the KSHV lytic cycle.

### Caspase activation is required for the proteolytic cleavage of Sp3 in the viral lytic cycle.

**FIG 2:**
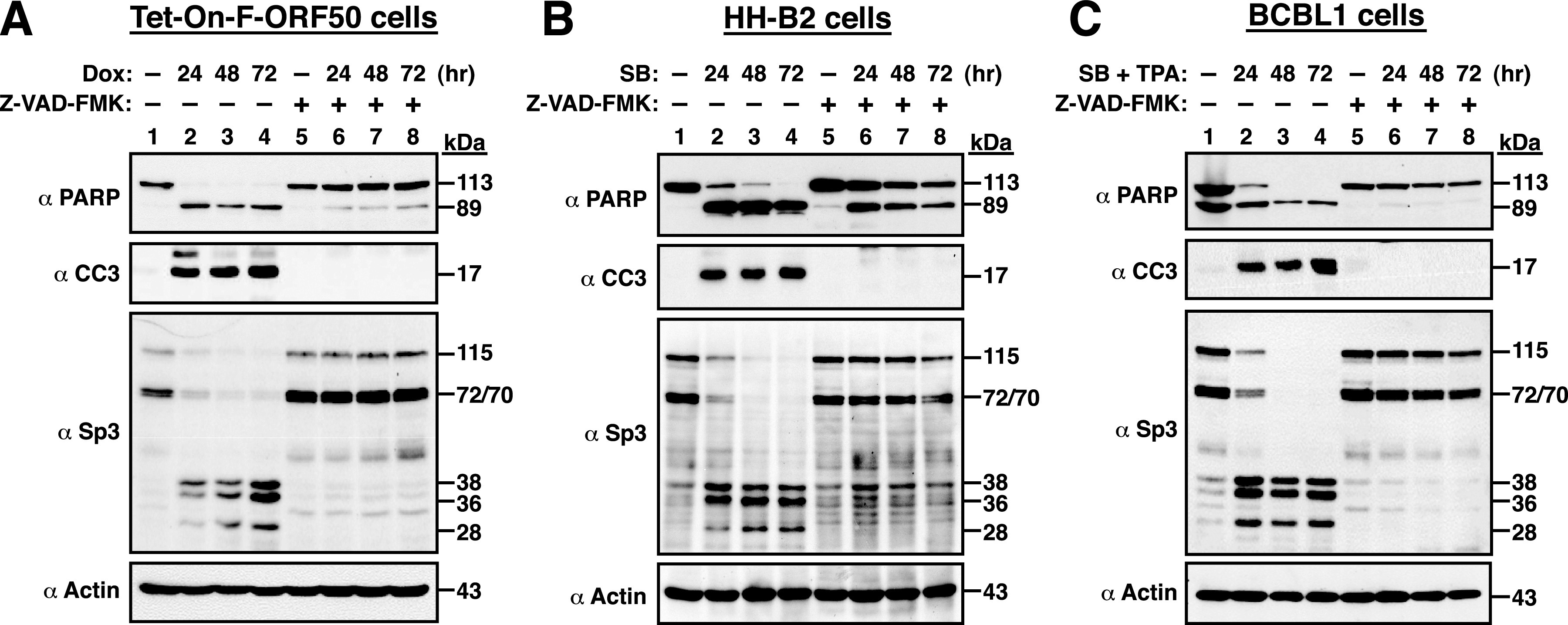
The proteolytic cleavage of Sp3 in the viral lytic cycle is dependent on caspase activation. PEL cells lines including Tet-On-F-ORF50 (A), HH-B2 (B), and BCBL-1 (C) were preincubated with or without a pan-caspase inhibitor Z-VAD-FMK (100 μM) for 1 h, followed by cotreatment with doxycycline, SB, and SB plus TPA, respectively. At the indicated time points (24, 48, and 72 h) after treatment, cells were prepared and analyzed for the expression of cleaved PARP, cleaved caspase-3 (CC3), and Sp3 by Western blotting. The untreated cell samples (lane 1) and cell samples receiving only Z-VAD-FMK (lane 5), which were harvested after 24 h of culture, served as control groups.

Since reactivation of KSHV or other human herpesviruses is often linked to caspase activation and apoptosis (31–39) and Sp3 is known as a substrate of caspases (30), we investigated whether the Sp3 processing observed in the viral lytic cycle was associated with caspase activation. To do this, PEL cells including Tet-On-F-ORF50, HH-B2, and BCBL1 were preincubated with or without a pan-caspase inhibitor Z-VAD-FMK for 1 h and then treated with the lytic cycle-inducing agents (Fig. 2A to C). At the indicated time points (24, 48, and 72 h) after treatment, Sp3 cleavage in these treated cells was examined by Western blotting. Treatment of these PEL cells with chemical inducers substantially triggered cellular apoptosis as detected by the production of cleaved poly(ADP-ribose) polymerase (cleaved PARP; 89 kDa) and cleaved caspase-3 (active form; 17 kDa) (Fig. 2A to C). However, incubation with Z-VAD-FMK (100 μM) significantly inhibited the cleavage of PARP and caspase-3 in Tet-On-HH-B2, HH-B2, and BCBL1 cells treated with doxycycline, SB, and the combination of SB and TPA, respectively (Fig. 2A to C). Under such conditions, the proteolytic cleavage of Sp3 in these chemical-treated PEL cells was also blocked by Z-VAD-FMK (Fig. 2A to C). These results strongly suggested that the proteolytic cleavage of Sp3 in the KSHV lytic cycle is dependent on caspase activation. During the course of experiments, we additionally noticed that besides the caspase-cleaved 89-kDa PARP fragment, there were another two cleaved PARP fragments with the molecular masses of 50 and 28 kDa detected in all PEL cell lines after treatment with inducing agents (see Fig. S1 in the supplemental material). Further studies may be needed to better characterize the 50- and 28-kDa PARP fragments. Furthermore, we found that untreated BCBL1 cells appeared to express relatively higher levels of cleaved PARP fragments than untreated HH-B2 cells (Fig. 2B and C), and treatment of BCBL1 cells with Z-VAD-FMK markedly reduced the overall level of PARP, especially its cleavage fragments (Fig. 2C, compare lane 1 to lane 5). Since almost all caspases are known to cleave PARP, it is possible that BCBL1 cells might have higher basal activity of caspases than HH-B2 cells under normal conditions.

### The proteolytic cleavage of Sp3 commonly occurs in both KSHV-positive and KSHV-negative cells that undergo apoptosis.

**FIG 3:**
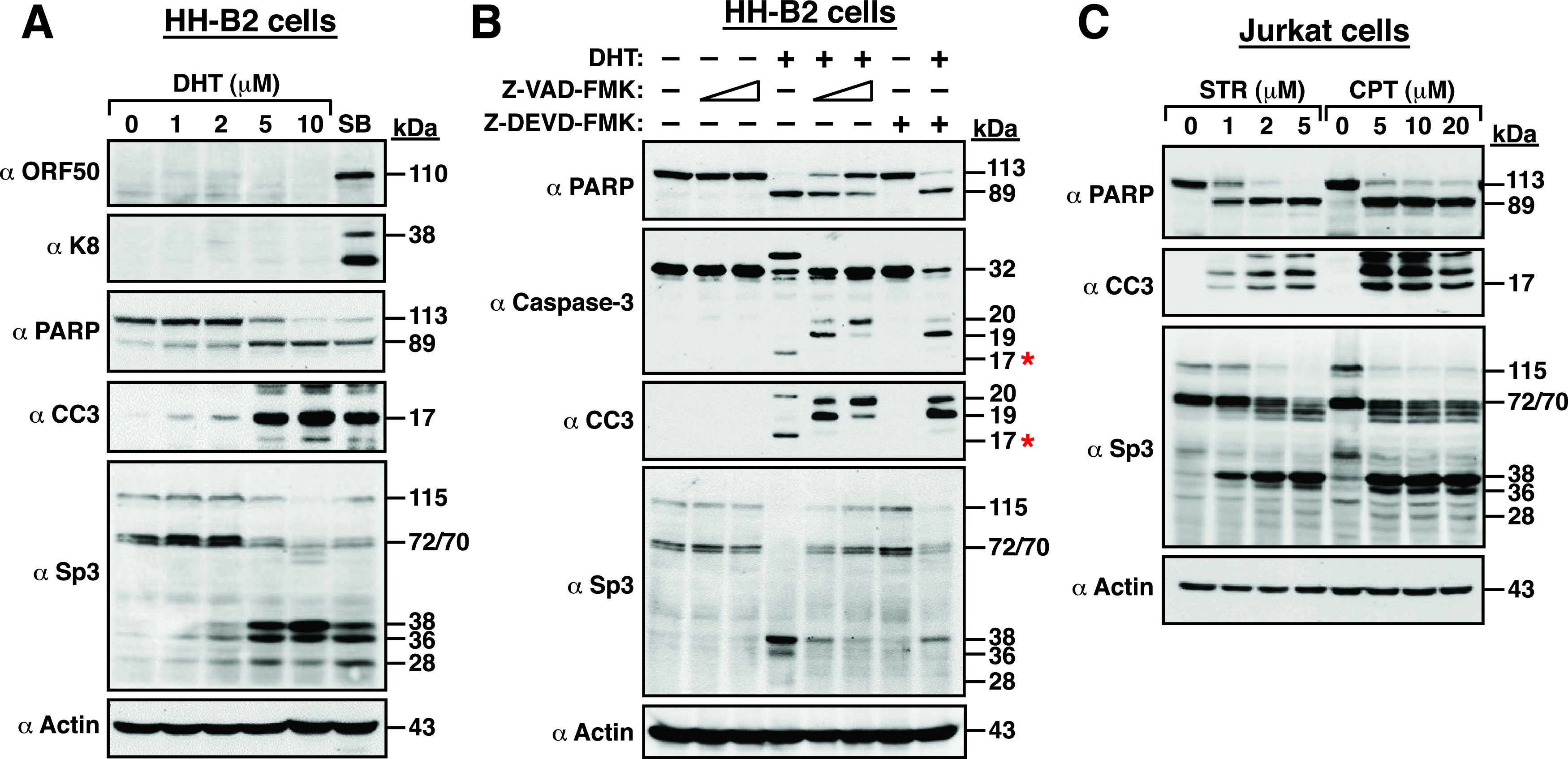
Both KSHV-positive and KSHV-negative cells treated with apoptosis-inducing agents elicit similar cleavage patterns of Sp3. (A) Induction of apoptosis in HH-B2 cells by dihydrotanshinone (DHT). HH-B2 cells were treated with increasing concentrations (0, 1, 2, 5, and 10 μM) of DHT for 24 h. Cell lysates were prepared and subjected to Western blot analysis for detecting the expression of ORF50, K8, Sp3, PARP, and cleaved caspase-3 (CC3) using specific antibodies. In parallel, HH-B2 cells treated with 3 mM sodium butyrate (SB) for 24 h were used as a positive control in the experiment. (B) Effects of a pan-caspase inhibitor Z-VAD-FMK or a caspase-3 inhibitor Z-DEVD-FMK on the DHT-mediated cleavage of Sp3. HH-B2 cells were pretreated with the pan-caspase inhibitor Z-VAD-FMK (50 or 100 μM) or the caspase 3 inhibitor Z-DEVD-FMK (200 μM) for 1 h and then cotreated with DHT (10 μM) for 24 h. Cell lysates were subjected to Western blot analysis for the detection of Sp3, PARP, caspase-3, and CC3 using specific antibodies. Red asterisks indicate the active form (p17, 17 kDa) of cleaved caspase-3. (C) Induction of apoptosis in Jurkat cells by staurosporine (STR) or camptothecin (CPT). Jurkat cells were treated with different concentrations (0, 1, 2, and 5 μM) of STR for 4 h or different concentrations (0, 5, 10, and 20 μM) of CPT for 6 h. After treatment, cells were harvested and analyzed for the expression of Sp3, PARP, and CC3 by Western blotting.

Next, we investigated whether induction of apoptosis (or caspase activation) in PEL cells was sufficient to promote the proteolytic cleavage of Sp3. We here used dihydrotanshinone (DHT), a major bioactive ingredient of Salvia miltiorrhiza, as an apoptotic inducer to treat HH-B2 cells because several studies have shown that DHT was able to trigger caspase- and reactive oxygen species (ROS)-dependent apoptosis in a variety of cancer cell types including B lymphoma cells (40–45). Treatment of HH-B2 cells with DHT significantly induced the cleavage of PARP and caspase-3 in a dose-dependent manner (Fig. 3A). However, unlike SB treatment, DHT treatment did not activate the expression of viral lytic proteins, such as ORF50 and K8 in HH-B2 cells (Fig. 3A). In addition to the classical replication program that is initiated by the expression of ORF50, Prasad et al. (35, 46) previously reported that KSHV also had an alternative apoptosis-initiated replication program characterized by a lack of the requirement for ORF50 protein, accelerated expression kinetics of late genes, and production of virus with low infectivity. Thus, it may be possible that DHT could trigger an alternative replication program of KSHV in HH-B2 cells via apoptosis. However, this notion needs to be further confirmed by experimental validation. Although DHT did not induce the expression of ORF50 and K8 in HH-B2 cells, the cleavage pattern of Sp3 in DHT-treated cells was similar to that in SB-treated cells (Fig. 3A). To confirm the involvement of caspases in Sp3 cleavage, cells were pretreated with the pan-caspase inhibitor Z-VAD-FMK or the caspases-3 inhibitor Z-DEVD-FMK for 1 h and then cotreated with DHT for 24 h. As shown in Fig. 3B, pretreatment with Z-VAD-FMK (50 or 100 μM) or Z-DEVD-FMK (200 μM) significantly prevented the DHT-mediated cleavage of caspase-3. Of note, several inactive caspase-3 precursors including p19 (19 kDa) and p20 (20 kDa) could be accumulated in DHT-treated cells in the presence of Z-VAD-FMK or Z-DEVD-FMK (Fig. 3B). Under such conditions, Z-VAD-FMK or Z-DEVD-FMK was also able to significantly attenuate DHT-mediated cleavage of Sp3 (Fig. 3B), suggesting that activation of caspases, particularly caspase-3, was critically involved in the proteolytic cleavage of Sp3 during apoptosis.

To examine the association between apoptosis and Sp3 cleavage in KSHV-negative cells, we used Jurkat cells, a human T-cell lymphoma cell line, as a model system. To induce cell apoptosis, Jurkat cells were treated with increasing concentrations of staurosporine (STR) for 4 h or camptothecin (CPT) for 6 h. STR is a protein kinase inhibitor (47), whereas CPT is known as a DNA topoisomerase I inhibitor (48). Following short-term treatment, we found that besides the cleavage of PARP and caspase-3, the proteolytic cleavage of Sp3 was also concomitantly detected in these treated cells (Fig. 3C). These results suggested that the proteolytic cleavage of Sp3 could be a common event in cells undergoing apoptosis or other situations in which caspases are activated. In the short-term treatment with STR or CPT, besides the three cleavage products (including 38, 36, and 28 kDa) of Sp3 as mentioned above, we also found a group of cleavage intermediates of Sp3 ranging from 60 to 70 kDa detected in these treated cells (Fig. 3C).

### Three major cleavage products of Sp3 detected in the KSHV lytic cycle contain the intact DNA-binding domain.

**FIG 4:**
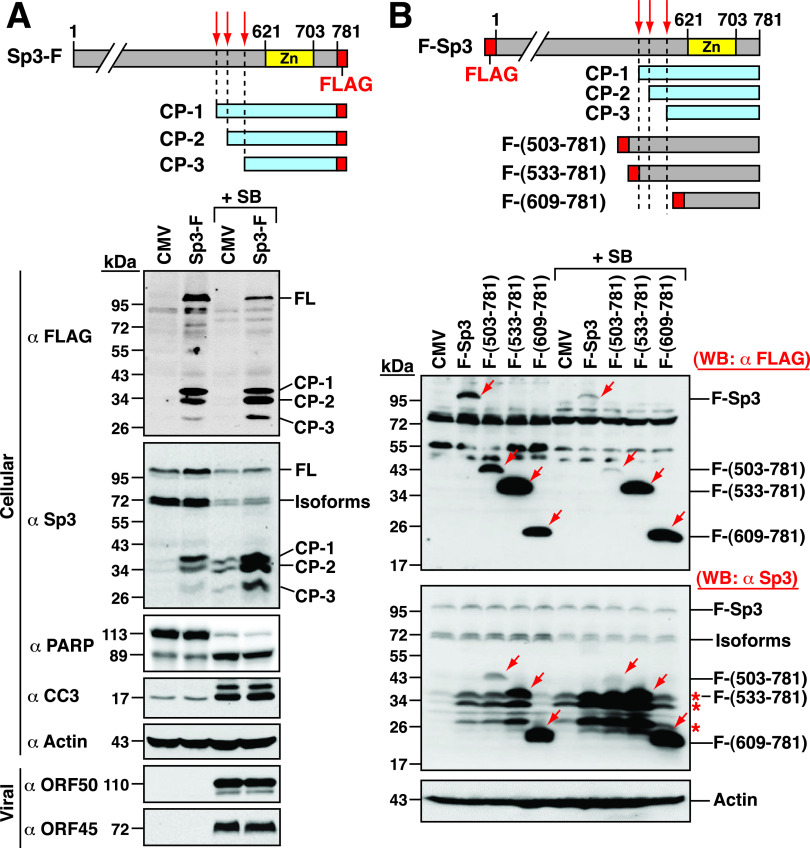
All three major cleavage products of Sp3, including CP-1, CP-2, and CP-3, possess an intact DNA-binding domain. (A) Western blot analysis of HH-B2 cells expressing the C-terminal FLAG-tagged Sp3 (Sp3-F). The upper panel shows a schematic diagram of the Sp3-F construct that contains a FLAG tag at the C-terminal end of Sp3. The zinc-finger DNA-binding domain located in aa 621 to 703 is also shown in the diagram. At 24 h after transfection and treatment with SB, the expression of Sp3-F in HH-B2 cells was analyzed by Western blotting using anti-FLAG or anti-Sp3 antibody. As noted, in addition to the full-length Sp3-F, three small Sp3-F fragments with the same migration as endogenous Sp3 cleavage products (CP-1, CP-2, and CP-3) were detected in the experiment using anti-Sp3 antibody. The cleavage of PARP and caspase-3 served as markers of apoptosis, whereas the expression of viral proteins ORF50 and ORF45 served as markers of the viral lytic reactivation. (B) Western blot analysis of HH-B2 cells expressing the N-terminal FLAG-tagged Sp3 (F-Sp3) or its deleted mutants, including F-Sp3(503-781), F-Sp3(533-781), or F-Sp3(609-781). The upper panel shows a schematic diagram of wild-type and mutant F-Sp3 constructs. The zinc-finger DNA-binding domain located in aa 621 to 703 is also shown in the diagram. HH-B2 cells were transfected with the indicated expression plasmids and then left untreated or treated with SB for 24 h. Cell lysates were prepared and subjected to Western blot analysis using anti-FLAG or anti-Sp3 antibody (bottom). Red arrows in Western blots indicate the positions of F-Sp3 or its deletion mutants, and red asterisks indicate the positions of endogenous Sp3 cleavage products (CP-1, CP-2, and CP-3).

As described above, three major cleavage products of Sp3, designated as CP-1 (38 kDa), CP-2 (36 kDa), and CP-3 (28 kDa), were routinely detected during KSHV reactivation in PEL cells. Since the anti-Sp3 antibody used in the study recognizes the epitope from aa 627 to 651 in Sp3, the three cleavage products would be generated from the C-terminal region of Sp3 and may cover the entire DNA-binding domain located between aa 621 and 703 (24). To map the regions of these Sp3 fragments, HH-B2 cells were transfected with the plasmid expressing the full-length Sp3 with a C-terminal FLAG tag (Sp3-F), and the transfected cells were left untreated or treated with SB. In the Sp3-F-transfected cells, besides the full-length Sp3-F (115 kDa), three cleavage products with the sizes of 38, 36, and 28 kDa were concurrently detected in Western blotting experiments using anti-FLAG antibody (Fig. 4A, α FLAG). Treatment with SB resulted in a reduced level of the full-length Sp3-F, along with increased production of these cleavage fragments (Fig. 4A, α FLAG). Similar results were also detected using anti-Sp3 antibody in Western blot analysis (Fig. 4A, α Sp3). Our data clearly show that the 38-, 36- and 28-kDa protein fragments of Sp3-F were corresponding to three endogenous cleavage products (CP-1, CP-2, and CP-3) of Sp3 (Fig. 4A). From the above experiment, it is worth mentioning that overexpression of Sp3-F did not significantly affect the cleavage of PARP and capase-3 in untreated or SB-treated HH-B2 cells. To estimate the coverage of endogenous Sp3 fragments, the N-terminal FLAG-tagged Sp3 (F-Sp3) or its N-terminal deletion mutants, including F-Sp3(503–781), F-Sp3(533–781), and F-Sp3(609–781), were expressed in HH-B2 cells (Fig. 4B). All of these F-Sp3 deletion constructs contain an intact DNA-binding domain. Western blot analysis revealed that all three endogenous Sp3 cleavage products (including CP-1, CP-2, and CP-3) were larger than F-Sp3(609–781) but smaller than F-Sp3(503–781). Furthermore, we found that the levels of F-Sp3, F-Sp3(503–781), and F-Sp3(533–781), but not F-Sp3(609–781), were significantly reduced in SB-treated cells compared to those of untreated cells (Fig. 4B, α FLAG), confirming that SB treatment could promote the proteolytic processing of F-Sp3, F-Sp3(503–781), and F-Sp3(533–781) but not F-Sp3(609–781). Based on these results, we concluded that the CP-1, CP-2, and CP-3 fragments were generated from the C-terminal region of Sp3, and all of them contain an intact DNA-binding domain (Fig. 4B).

### Potential caspase cleavage sites in Sp3.

**FIG 5:**
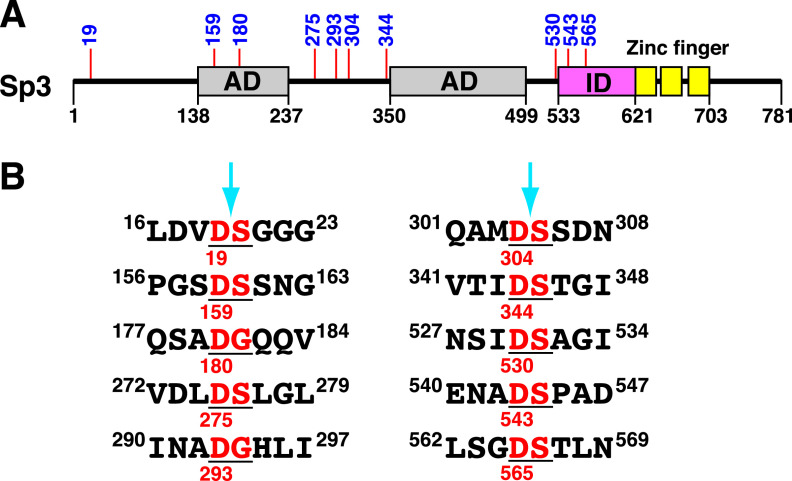
Potential caspase cleavage sites in Sp3. (A) Schematic diagram of the functional domains and the potential caspase cleavage sites in Sp3. The full-length Sp3 contains two glutamine-rich transactivation domains (AD; aa 138–237 and aa 350–499), a transactivation-inhibitory domain (ID; aa 533–621), and a DNA-binding domain consisting of three Cys2-His2 zinc fingers (aa 621–703). Numbers above the diagram represent the positions of the critical aspartate residues in the potential caspase cleavage sites. (B) List of the potential caspase cleavage sites containing a D–G or D–S motif in Sp3.

Caspases are cysteine-aspartic proteases that specifically cleave a substrate protein immediately following aspartate (D) residues. Ample studies have shown that caspases preferentially recognize and cleave the consensus DEXD–(G/S) or XEXD–(G/S) motif (where X is any amino acid and “–” denotes the cleavage site) (49, 50). However, no canonical caspase cleavage sites could be found in Sp3 by a web-based prediction tool, PeptideCutter (https://web.expasy.org/peptide_cutter/). Despite a lack of the consensus caspase cleavage sites in Sp3, we sought to examine all sites that contain a D–G or D–S motif within Sp3. There are 10 D–(G/S) motifs in Sp3, and the critical aspartate residues are situated at positions 19, 159, 180, 275, 293, 304, 344, 530, 543, and 565 (Fig. 5A). The amino acid sequences of these 10 potential caspase cleavage motifs are listed in Fig. 5B. In fact, when we attempted to predict the caspase cleavage sites in Sp3 by another prediction tool (SitePrediction; https://www.dmbr.ugent.be/prx/bioit2-public/SitePrediction/), we found that all of our predicted sites listed in Fig. 5B are included in the prediction results returned by SitePrediction (see Fig. S2 in the supplemental material).

### Mapping of the caspase cleavage sites in the C-terminal region of Sp3.

**FIG 6:**
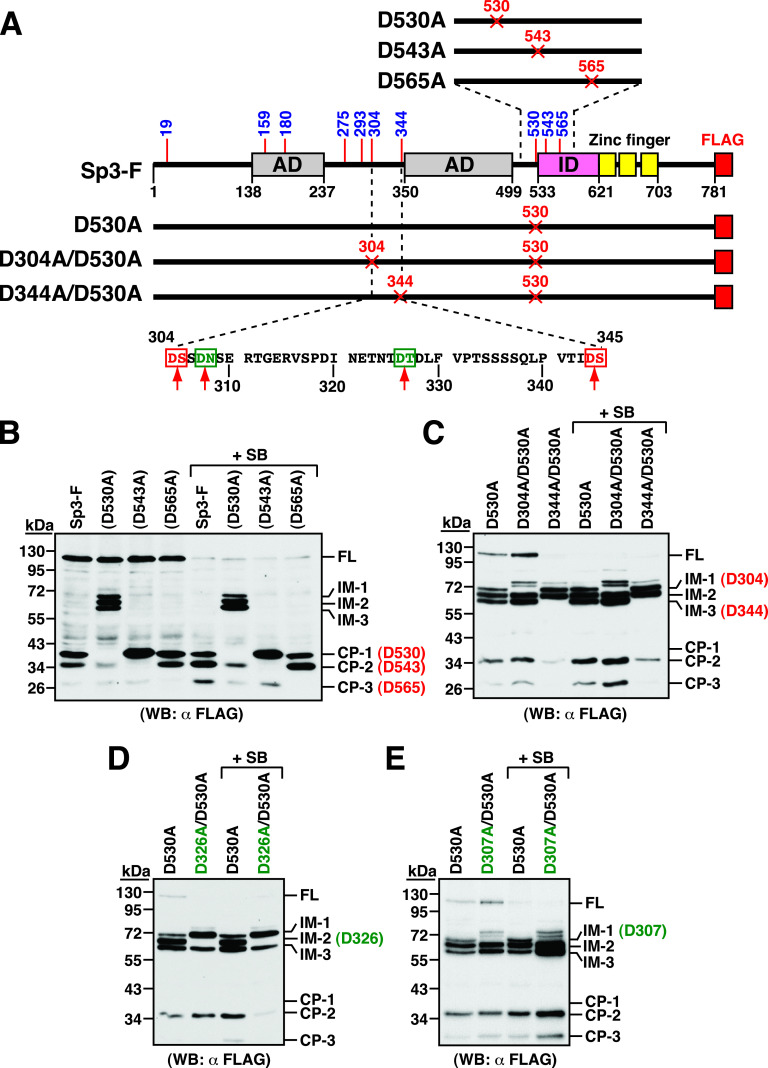
Mapping of the caspase cleavage sites in the C-terminal region of Sp3. (A) Diagram of wild-type and mutant Sp3-F constructs. AD, activation domains; ID, transactivation-inhibitory domain; zinc finger, DNA-binding domain. Red amino acids depicted in the diagram represent the potential caspase cleavage sites that are included in the original prediction, whereas green amino acids in the diagram represent the potential caspase cleavage sites that are not included in our original prediction. (B) Western blot analysis of wild-type Sp3-F and the mutant proteins containing Sp3-F(D530A), Sp3-F(D543A), and Sp3-F(D565A). The expression plasmids encoding wild-type or mutant Sp3-F proteins were individually transfected into HH-B2 cells in the presence or absence of SB. At 24 h after transfection and treatment, cells were harvested and subjected to Western blot analysis to evaluate the cleavage pattern of Sp3-F and its mutant proteins using anti-FLAG antibody. Three cleavage products including CP-1, CP-2, and CP-3 were differentially detected in different Sp3-F mutants, and three longer cleavage intermediates (IM-1, IM-2, and IM-3) were specifically detected in Sp3-F(D530A). (C) Western blot analysis of Sp3-F mutant constructs containing Sp3-F(D530A), Sp3-F(D304A/D530A), and Sp3-F(D344A/D530A). The cleavage pattern of Sp3-F mutants expressed in HH-B2 cells was analyzed by Western blotting using anti-FLAG antibody. Notably, the cleavage intermediates IM-1 and IM-3 were corresponding to the Sp3 C-terminal fragments generated by cutting at D304 and D344, respectively. (D) Western blot analysis of Sp3-F mutants containing Sp3-F(D530A) and Sp3-F(D326A/D530A). Note that the Sp3-F(D326A/D530A) construct failed to express the IM-2 fragment. (E) Western blot analysis of Sp3-F mutants containing Sp3-F(D530A) and Sp3-F(D307A/D530A). Note that the Sp3-F(D307A/D530A) construct had a defect in the production of the IM-1 fragment.

Based on the Western blot results shown in Fig. 4, the cleavage sites for generating the CP-1 (38 kDa), CP-2 (36 kDa), and CP-3 (28 kDa) fragments from Sp3 were located between aa 503 and 609. To determine the cleavage sites that generate CP-1, CP-2, and CP-3, the predicted amino acid residues including D530, D543, and D565 were individually mutated to alanine (A) in Sp3-F (Fig. 6A). The resulting constructs, Sp3-F(D530A), Sp3-F(D543A), and Sp3-F(D565A), were transfected into HH-B2 cells, and then the transfected cells were left untreated or treated with SB for 24 h. As shown in Fig. 6B, we failed to detect the CP-3 fragment from Sp3-F(D565A) and the CP-2 fragment from Sp3-F(D543A), suggesting that the residues D543 and D565 were the cleavage positions that generate CP-2 and CP-3, respectively. Intriguingly, compared to wild-type Sp3-F, the Sp3-F(D530A) construct had several unique features in HH-B2 cells, which included (i) lacking the CP-1 fragment, (ii) generating three additional cleavage intermediates ranging from 60 to 70 kDa (designated as IM-1, IM-2, and IM-3), and (iii) exhibiting reduced levels of both CP-2 and CP-3 fragments (Fig. 6B). These results implied that the CP-1 fragment, which was generated through cleavage at D530, could be a major precursor of CP-2 and CP-3.

Due to the stable production of IM-1 (70 kDa), IM-2 (66 kDa), and IM-3 (62 kDa) from Sp3-F(D530A), we extended our studies to map the cleavage sites of these intermediates. According to the predicted sizes, the residues D304 and D344 were chosen to be mutated into alanine in Sp3-F(D530A) (Fig. 6A). The resulting constructs, Sp3-F(D304A/D530A) and Sp3-F(D344A/D530A), were then individually expressed in HH-B2 cells. Western blot analysis showed that the IM-1 and IM-3 fragments could not be produced from Sp3-F(D304A/D530A) and Sp3-F(D344A/D530A), respectively (Fig. 6C), indicating that the residues D304 and D344 were the cleavage positions that generate IM-1 and IM-3, respectively. To further map the caspase cleavage site for IM-2, the region between aa 304 and 344 was analyzed, and three candidate sites were predicted at D319, D326, and D328 (Fig. 6A). Mutational analysis showed that only introduction of D326A substitution in Sp3-F(D530A) led to a defect in the generation of IM-2 (Fig. 6D; data not shown). Besides these aspartate residues, we also mutated the amino acid residue D307 in Sp3-F(D530A). Intriguingly, we found that similar to Sp3-F(D304A/D530A), the resulting construct Sp3-F(D307A/D530A) also showed a defect in the generation of IM-1 (Fig. 6E), suggesting that both D304 and D307 were part of the core element in the same caspase cleavage motif. Taken together, our data suggested that the residues D304 (or D307), D326, and D344 were the cleavage positions that generate IM-1, IM-2, and IM-3, respectively.

### Mapping of the caspase cleavage sites in the N-terminal region of Sp3.

**FIG 7:**
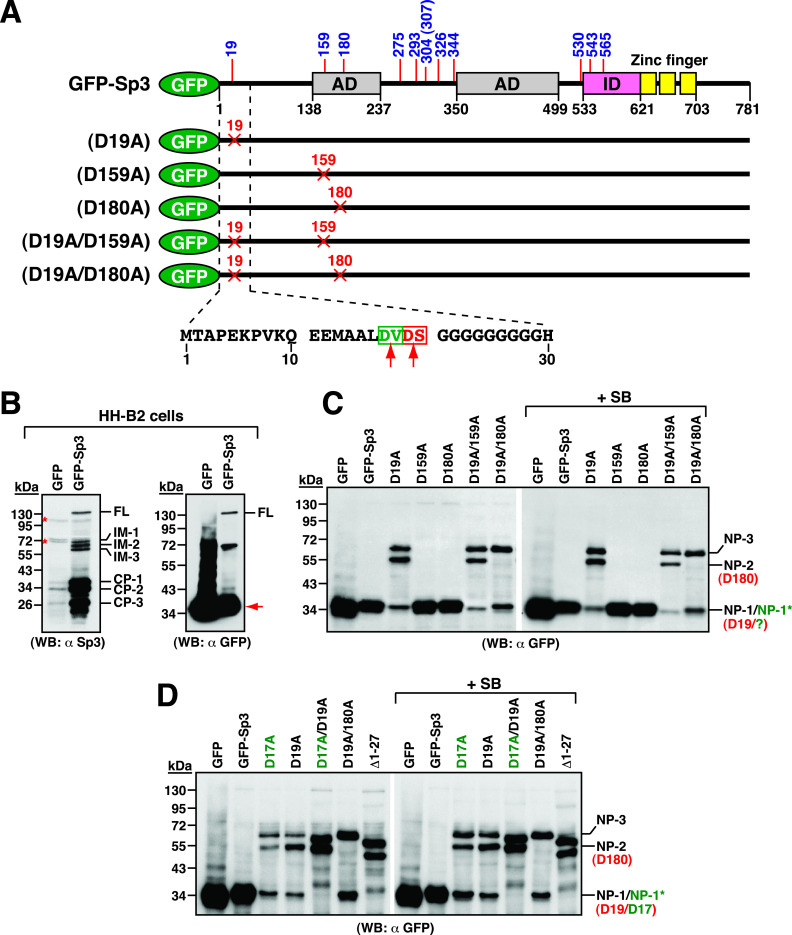
The amino acid positions at D17, D19, and D180 in the N-terminal region of Sp3 are identified as the caspase cleavage sites. (A) Diagram of GFP-Sp3 and its mutant constructs containing D19A, D159A, D180A, D19A/D159A, or D19A/D180A substitution. Two potential caspase cleavage sites, D17 (green color) and D19 (red color), within the region between aa 1 and 30 are illustrated in the diagram. (B) Proteolytic cleavage of the GFP-Sp3 fusion construct in HH-B2 cells. The wild-type GFP-Sp3 construct expressed in HH-B2 cells was analyzed by Western blotting using anti-Sp3 or anti-GFP antibody. Multiple cleavage products of GFP-Sp3, including CP-1, CP-2, CP3, IM-1, IM-2, and IM-3, could be detected in Western blot analysis using anti-Sp3 antibody, whereas an abundant cleavage product with a size of 36 kDa (red arrowhead) could be detected using anti-GFP antibody. (C) Western blot analysis of the cleavage pattern of GFP-Sp3 mutants. The indicated expression plasmids were individually transfected into HH-B2 cells in the presence or absence of SB for 24 h, and the cleavage pattern of GFP-Sp3 mutants was analyzed by Western blotting using anti-GFP antibody. Notably, at least three N-terminal cleavage fragments (NP-1/NP-1*, NP-2, and NP-3) could be differentially generated from these GFP-Sp3 mutants. (D) Characterization of the NP-1* fragment from GFP-Sp3. The GFP-Sp3 mutant constructs containing D17A substitution or the N-terminal 27-aa deletion were analyzed for the cleavage pattern in HH-B2 cells by Western blotting using anti-GFP antibody.

To identify the caspase cleavage sites near the N-terminal region of Sp3, we tagged the green fluorescent protein (GFP) to the N terminus of Sp3 to generate a fusion construct GFP-Sp3 (Fig. 7A). When GFP-Sp3 was expressed in HH-B2 cells, three C-terminal cleavage products (CP-1, CP-2, and CP-3) as well as three larger intermediates (IM-1, IM-2, and IM-3) could be concurrently detected in Western blot analysis using anti-Sp3 antibody (Fig. 7B). When the same protein lysates were probed with anti-GFP antibody, a major cleavage product with the size of 36 kDa was observed (Fig. 7B, α GFP). It was worth noting that GFP-Sp3 expressed in 293T cells was cleaved only slightly under normal conditions (data not shown), implicating that the basal caspase activity in HH-B2 cells might be higher than that in 293T cells.

We next mutated the residues at D19, D159, or D180 to alanine in GFP-Sp3 (Fig. 7A). As shown in Fig. 7C, only GFP-Sp3(D19A), but not GFP-Sp3(D159A) and GFP-Sp3(D180A), in HH-B2 cells displayed an abnormal cleavage pattern showing that the 36-kDa GFP-tagged Sp3 fragment was dramatically reduced, resulting in the production of two longer GFP-tagged Sp3 fragments (55 and 66 kDa). These results suggested that the residue D19 in Sp3 was the caspase cleavage position, and mutation of this residue led to the production of larger fragments. For the sake of convenience, the GFP-tagged Sp3 fragments including 36, 55, and 66 kDa were designated as NP-1, NP-2, and NP-3, respectively (Fig. 7C). To further map the cleavage positions for NP-2 and NP-3, we introduced substitution of D159A or D180A in GFP-Sp3(D19A) (Fig. 7A). Western blot analysis revealed that GFP-Sp3(D19A/D180A), but not GFP-Sp3(D19A/D159A), exhibited a defect in producing the NP-2 fragment (Fig. 7C), suggesting that the residue D180 was the cleavage position that generates NP-2.

Since a minor band with a similar size to NP-1 was consistently detected from GFP-Sp3(D19A) or its derivative constructs (Fig. 7C), we then attempted to characterize the protein fragment (designated as NP-1*). The NP-1* fragment might be equivalent to “pure GFP” occasionally falling off from the GFP fusion proteins during the process of total protein extraction from cells (such as heating treatment or sonication), or it might be generated by cutting at a specific site in the extreme N-terminal region of Sp3. In the latter case, the residue D17 located in the N-terminal region was a candidate site (Fig. 7A). As shown in Fig. 7D, the mutant GFP-Sp3(D17A) displayed a cleavage pattern similar to that of GFP-Sp3(D19A), whereas the double-point mutant GFP-Sp3(D17A/D19A) failed to produce the 36-kDa fragments corresponding to NP-1/NP-1*. These results suggested that the residue D17 was a cleavage site that generates NP-1*. To further confirm our findings, we deleted the N-terminal 27-aa region of Sp3 in GFP-Sp3 and assayed the cleavage pattern of the deletion mutant GFP-Sp3(Δ1-27) in HH-B2 cells (Fig. 7D). Our results indeed demonstrated that GFP-Sp3(Δ1-27) could not produce the 36-kDa band that is equivalent to NP-1/NP-1* (Fig. 7D).

**FIG 8:**
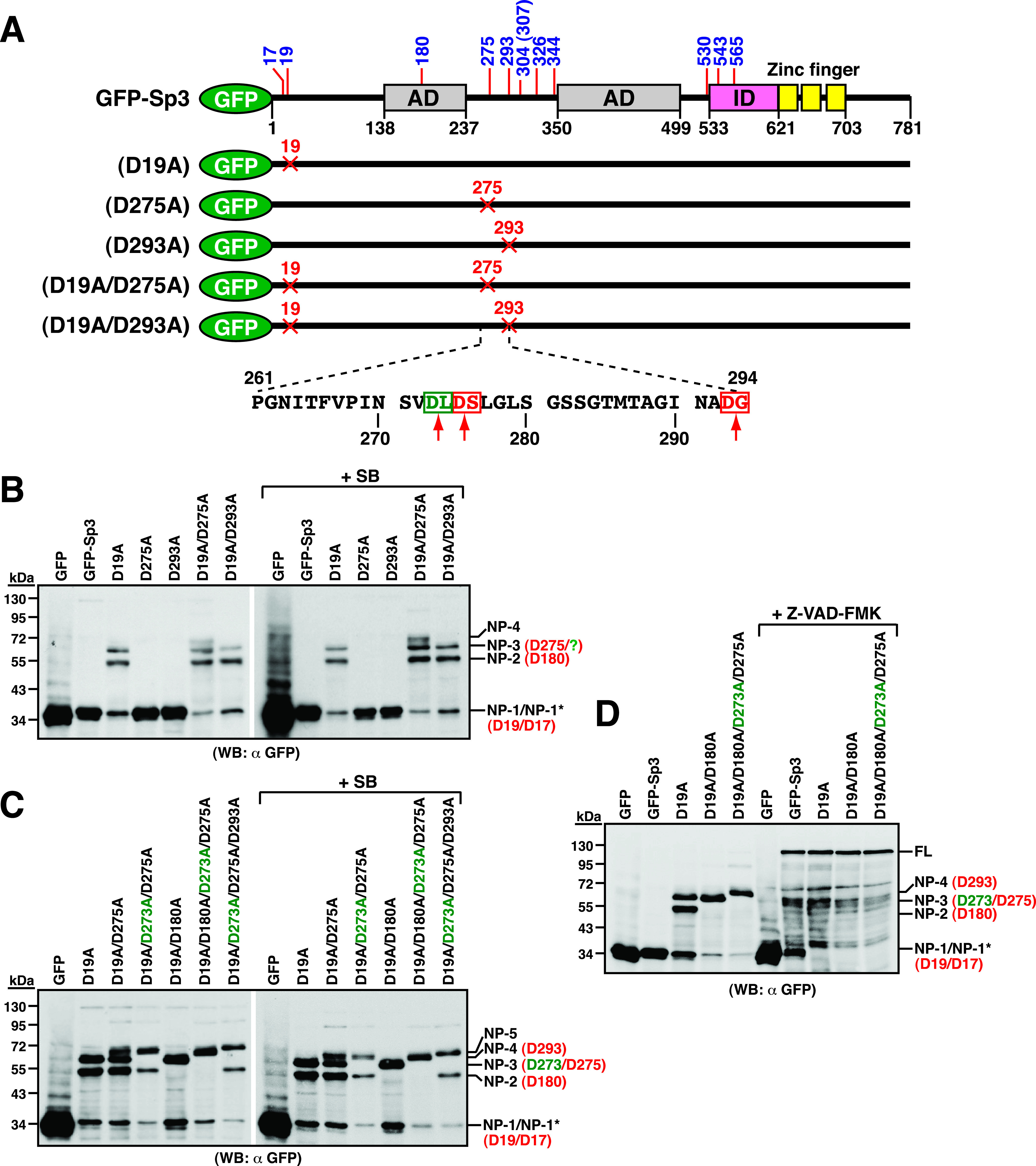
The amino acid residues including D273, D275, and D293 in Sp3 are identified as the caspase cleavage sites. (A) Diagram of wild-type GFP-Sp3 and the mutant constructs containing D19A, D275A, D293A, D19A/D275A, and D19A/D293A substitution. Two potential caspase cleavage sites, D273 (green color) and D275 (red color), in the region between aa 261 and 294 are shown in the diagram. (B) Western blot analysis of GFP-Sp3 mutant proteins containing GFP-Sp3(D19A/D275A) and GFP-Sp3(D19A/D293A). The indicated expression plasmids were individually transfected into HH-B2 cells in the presence or absence of SB for 24 h. Cell lysates were analyzed by Western blot analysis to examine the proteolytic cleavage of these mutant constructs using anti-GFP antibody. Of note, in addition to NP-1/NP-1*, NP-2, and NP-3, an extra slow-migrating band (NP-4) could be produced from GFP-Sp3(D19A/D275A). (C) Western blot analysis of GFP-Sp3 mutant proteins containing GFP-Sp3(D19A/D273A/D275A) and GFP-Sp3(D19A/D273A/D275A/D293A). The indicated expression plasmids were individually transfected into HH-B2 cells in the presence or absence of SB for 24 h, and then the cleavage pattern of different GFP-Sp3 mutants was analyzed by Western blotting using anti-GFP antibody. Note that the “NP-3” band observed in the GFP-Sp3(D19A) could be completely shifted to “NP-4” after induction of the D273A/D275A substitution. Further introduction of D293A substitution into GFP-Sp3(D19A/D273A/D275A) resulted in a shift of “NP-4” to “NP-5.” (D) Effect of a pan-caspase inhibitor Z-VAD-FMK on the proteolytic cleavage of GFP-Sp3 and its mutants. The indicated expression plasmids were individually transfected into HH-B2 cells in the presence or absence of Z-VAD-FMK treatment. Western blot analysis was performed to determine the proteolytic cleavage of GFP-Sp3 and its mutants.

To characterize the NP-3 fragment (66 kDa) generated from GFP-Sp3(D19A), the potential cleavage site D275 or D293 was mutated in GFP-Sp3(D19A) (Fig. 8A). As compared to GFP-Sp3(D19A), there was no appreciable change in the cleavage pattern for GFP-Sp3(D19A/D293A) (Fig. 8B). Although GFP-Sp3(D19A/D275A) still generated the NP-3 fragment, an extra band (NP-4; 70 kDa) that migrated slower than the NP-3 band could be concurrently generated (Fig. 8B). These results implicated that the NP-3 band might contain more than one cleavage fragment, and one of them was equivalent to the protein fragment generated through cleavage at D275. To find the cleavage positions for other fragments in NP-3, we analyzed the amino acid sequence around D275 and found a potential candidate site at D273 (Fig. 8A). A triple-point mutant GFP-Sp3(D19A/D273A/D275A) was therefore constructed. Compared to GFP-Sp3(D19A) or GFP-Sp3(D19A/D275A), the triple-point mutant GFP-Sp3(D19A/D273A/D275A) failed to produce the NP-3 fragment but generated a larger fragment (NP-4; 70 kDa) (Fig. 8C). Consistently, the NP-3 band was also shifted to the NP-4 band in a quadruple-point mutant GFP-Sp3(D19A/D180A/D273A/D275A) compared to GFP-Sp3(D19A/D180A) (Fig. 8C). To further map the cleavage position of the NP-4 fragment, the residue D293 was mutated in GFP-Sp3(D19A/D273A/D275A). After introduction of D293A substitution in GFP-Sp3(D19A/D273A/D275A), the NP-4 band was shifted to a 72-kDa product (Fig. 8C, NP-5). Collectively, our studies identified that D273 and D275 were the two cleavage sites for NP-3, whereas D293 was the cleavage site for NP-4.

To demonstrate whether the identified cleavage sites in Sp3 were indeed associated with caspases, the pan-caspase inhibitor Z-VAD-FMK was used to treat cells that expressed wild-type GFP-Sp3 or its mutant constructs, including GFP-Sp3(D19A), GFP-Sp3(D19A/D180A), and GFP-Sp3(D19A/D180A/D273A/D275A). Our results clearly showed that treatment with Z-VAD-FMK markedly blocked the cleavage of the identified aspartate residues in Sp3 (Fig. 8D).

### Some cleavage products of Sp3 can cooperate with ORF50 to synergistically activate specific gene promoters.

**FIG 9:**
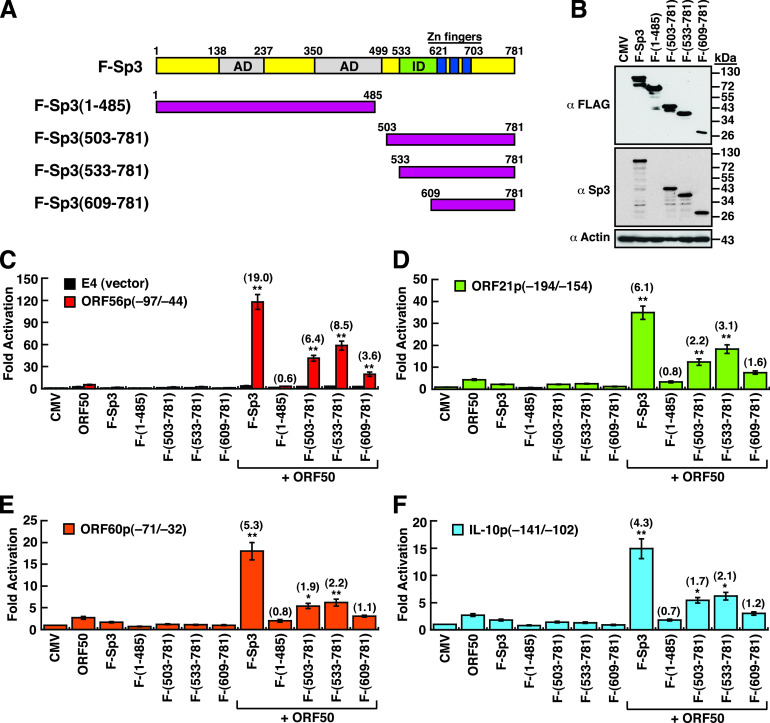
The Sp3 DNA-binding domain is essential for the synergy with ORF50 to activate specific viral and cellular gene promoters. (A) Schematic diagram of construction of the wild-type and mutant F-Sp3 proteins. AD, activation domains; ID, transactivation-inhibitory domain; zinc fingers, DNA-binding domain. (B) Western blot analysis of the wild-type and mutant F-Sp3 proteins expressed in 293T cells. It is worth noting that the anti-Sp3 antibody used in Western blotting recognizes the C-terminal epitope between aa 627 and 651. (C to F) Transcriptional activation of the ORF56p(−97/−44), ORF21p(−194/−154), ORF60p(−71/−32), or IL-10p(−141/−102) reporter construct by ORF50 in combination with different F-Sp3 mutants. Numbers in parenthesis indicate the synergistic indexes that were calculated as the ratio of the luciferase activity of the reporter construct in the presence of both ORF50 and specific F-Sp3 mutants over the sum of the activity of the reporter construct alone and the increases in this activity caused by the expression of ORF50 and specific F-Sp3 mutants individually. *, *P < *0.05; **, *P < *0.01, for results compared to those with the control pE4-luc reporter (Student's *t* test; *n *=* *3).

To study the potential impact of cleaved Sp3 fragments on ORF50-mediated transactivation, ORF50 and/or different F-Sp3 deletion constructs (Fig. 9A and B) were cotransfected with a set of the promoter/reporter plasmids into HKB5/B5 cells, and then the luciferase reporter activation was measured (Fig. 9C to F). Four promoter/reporter constructs that individually contain the ORF50 response elements, ORF56p(−97/−44), ORF21p(−194/−154), ORF60p(−71/−32), and IL-10p(−141/−102), were used in the study. Compared to the full-length F-Sp3, the mutant lacking the C-terminal DNA-binding domain, F-Sp3(1–485), completely lost the ability to cooperate with ORF50 to synergistically activate these reporter constructs (Fig. 9C to F). Surprisingly, three N-terminal deletions including F-Sp3(503–781), F-Sp3(533–781), and F-Sp3(609–781), which contain intact DNA-binding domains, still retained substantial ability for the synergy with ORF50 to activate the ORF56p(−97/−44) reporter construct, although their abilities were lower than that of full-length F-Sp3 (Fig. 9C). For the ORF21p(−194/−154), ORF60p(−71/−32), or IL-10p(−141/−102) reporter construct, the mutants F-Sp3(503–781) and F-Sp3(533–781), but not F-Sp3(609–781), still exhibited significant synergy with ORF50 on activation of these reporter constructs (Fig. 9D to F). These results suggest that specific cleavage products generated from Sp3 could play critical roles in the KSHV lytic cycle.

### The DNA-binding domain of Sp3 is involved in the interaction between ORF50 and the target promoter.

**FIG 10:**
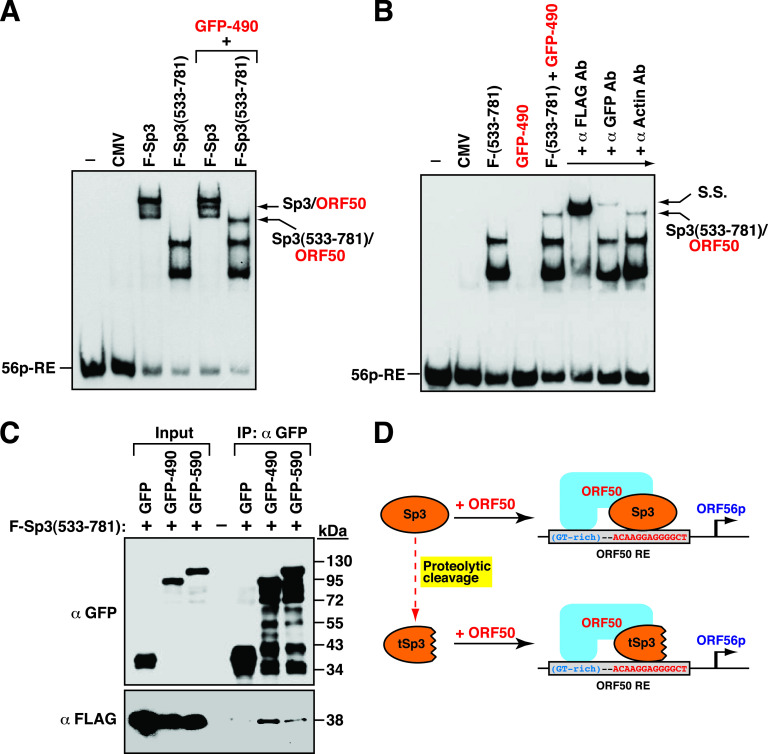
The Sp3 DNA-binding domain is required for recruiting ORF50 protein to the target DNA. (A) EMSA showing the Sp3/ORF50 protein complexes on the 56p-RE element. The EMSA gel image shown is representative of two independent experiments. In the EMSA, total protein extracts of 293T cells transfected with the full-length F-Sp3, F-Sp3(533-781), F-Sp3 plus GFP-ORF50(1-490), or F-Sp3(533-781) plus GFP-ORF50(1-490) constructs used. The ORF50-responsive element from the ORF56 gene promoter, 56p-RE, was used as a DNA probe. The F-Sp3/GFP-ORF50(1–490) and F-Sp3(533–781)/GFP-ORF50(1–490) protein complexes are indicated by arrows. (B) Antibody supershift testing in EMSA. The EMSA gel image shown is representative of two independent experiments. Antibodies against FLAG, GFP, or actin were used in the supershift study. S.S., supershifted complex. (C) Coimmunoprecipitation analysis of the interaction between F-Sp3(533–781) and GFP-ORF50(1–490) or GFP-ORF50(1–590). The F-Sp3(533–781) expression plasmid was cotransfected with the plasmids expressing GFP, GFP-ORF50(1–490), or GFP-ORF50(1–590) into 293T cells. At 24 h after transfection, cell lysates were prepared and then immunoprecipitated with anti-GFP antibody. The resultant immunoprecipitates were subjected to Western blot analysis using anti-GFP or anti-FLAG antibody. (D) Model for the assembly of ORF50 and Sp3 or truncated Sp3 on the ORF50-responsive element of the ORF56 promoter.

As describe above, F-Sp3(533–781), which is equivalent to the CP-1 fragment, retained the ability to cooperate with ORF50 to synergistically activate target gene promoters (Fig. 9). To determine whether F-Sp3(533–781) could recruit ORF50 to the target gene promoter, electrophoretic mobility shift assay (EMSA) experiments were performed using the ORF56p(−97/−44) element (designated as 56p-RE) as a DNA probe and different protein lysates from 293T cells transfected with F-Sp3, F-Sp3(533–781), GFP-ORF50(1–490), F-Sp3 plus GFP-ORF50(1–490), or F-Sp3(533–781) plus GFP-ORF50(1–490) (Fig. 10A and B). GFP-ORF50(1–490) is a GFP fusion construct containing the ORF50 DNA-binding domain from aa 1 to 490. Results from EMSAs showed that there were two major protein/DNA complexes detected using protein lysates containing either F-Sp3 or F-Sp3(533–781) alone (Fig. 10A). When the protein lysates containing F-Sp3 plus GFP-ORF50(1–490) or F-Sp3(533–781) plus GFP-ORF50(1–490) were used in EMSAs, an extra protein/DNA complex, corresponding to F-Sp3/ORF50/DNA or F-Sp3(533–781)/ORF50/DNA, could be detected (Fig. 10A). Of note, GFP-ORF50(1–490) alone could not bind the 56p-RE probe (Fig. 10B). In the EMSA supershift assay using anti-FLAG or anti-GFP antibody, we clearly demonstrated that both F-Sp3(533–691) and GFP-ORF50(1–490) could form a protein complex on the 56p-RE probe (Fig. 10B). To further verify the interaction between F-Sp3(533–781) and ORF50, a coimmunoprecipitation assay was performed. The protein lysates of 293T cells that were cotransfected with F-Sp3(533–781) and GFP, GFP-ORF50(1–490), or GFP-ORF50(1–590) were used here (Fig. 10C). After immunoprecipitation with anti-GFP antibody, we found that F-Sp3(533–781) could be coimmunoprecipitated with GFP-ORF50(1–490) or GFP-ORF50(1–590) but not GFP (Fig. 10C). Together, these results suggested that besides the full-length Sp3, some truncated Sp3 fragments could still retain the ability to recruit ORF50 to target DNA (Fig. 10D).

## DISCUSSION

Many viruses have evolved different strategies to utilize cellular components to favor their viral gene expression and survival. In the case of KSHV, ample evidence has shown that ORF50 protein is capable of activating its downstream target genes through several distinct mechanisms (15, 16). We have previously demonstrated that ORF50 can cooperate with Sp3 to synergistically activate a set of viral and cellular targets (23). Herein, we show that Sp3 actually undergoes proteolytic cleavage during the KSHV lytic cycle (Fig. 1). Despite the proteolytic cleavage of Sp3 in the viral lytic cycle, we find that several Sp3 fragments that contain intact DNA-binding domain potentially have a substantial ability for the synergy with ORF50 to activate the ORF56p(−97/−44), ORF21p(−194/−154), ORF60p(−71/−32), and IL10p(−142/−102) reporter constructs, albeit at a lower ability than wild-type Sp3 (Fig. 9). Of note, most of viral replication enzymes, including ORF56 (primase), ORF21 (thymidine kinase), and ORF60 (ribonucleotide reductase, small subunit), are thought to be expressed at relatively lower levels than other viral lytic proteins. We speculate that cooperation between ORF50 and the resulting Sp3 C-terminal fragments (Fig. 10 A to D) during the viral lytic cycle may be capable of inducing sufficient amounts of these viral enzymes for viral DNA replication.

Sp3 is a ubiquitously expressed transcription factor and is involved in multiple cellular processes (24–26). Actually, many previous studies have reported that apoptosis induced by various chemotherapeutic agents in a variety of cancer cells are often associated with “downregulation of Sp3” (detected by Western blot analysis) (51–56). Since most previous studies used only a single anti-Sp3 antibody (recognizing a particular region on Sp3) in Western blot experiments and mainly focused on the known isoforms of Sp3, several small cleaved fragments of Sp3 produced in apoptotic cells might be ignored or could not be detected by the antibodies used in their studies. Therefore, the occurrence of the caspase-mediated cleavage of Sp3 in apoptotic cells may be underestimated in previous studies.

**FIG 11:**
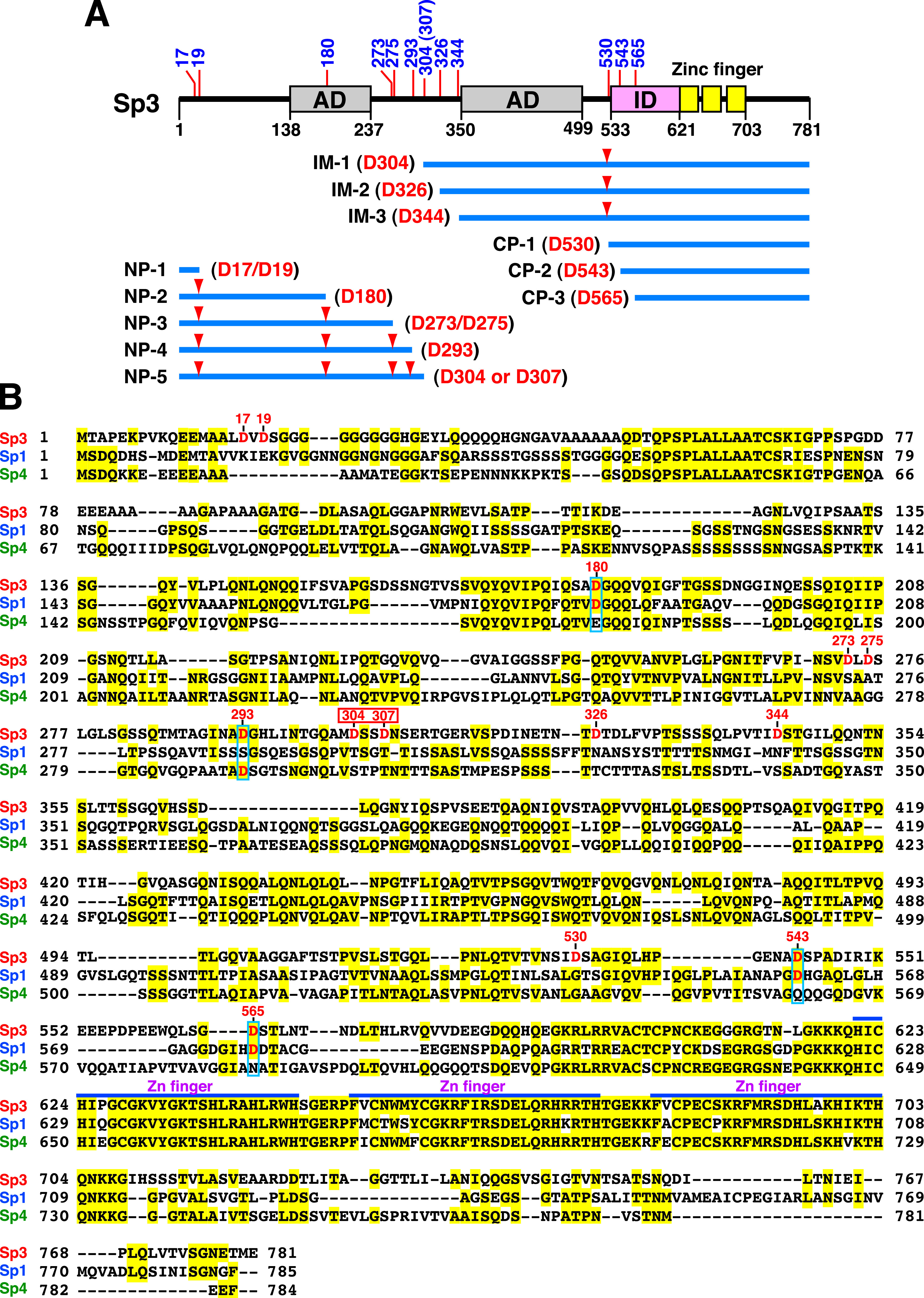
Summary of the caspase cleavage sites identified in Sp3 and comparison of the potential caspase cleavage sites among the Sp family proteins. (A) Schematic diagram of the functional domains and the caspase cleavage sites of Sp3. Twelve caspase cleavage positions in Sp3 and all possible cleavage fragments of Sp3 are shown in the diagram. AD, activation domains; ID, transactivation-inhibitory domain; zinc finger, DNA-binding domain. (B) Alignment of amino acid sequences of Sp3, Sp1, and Sp4. The protein sequence alignment of Sp3 (RefSeq accession number NP_003102.1), Sp1 (RefSeq accession number NP_612482.2), and Sp4 (RefSeq accession number NP_003103.2) was drawn using an alignment tool (COBALT; https://www.ncbi.nlm.nih.gov/tools/cobalt/re_cobalt.cgi). The critical aspartate (D) residues of the caspase cleavage sites in Sp3 are indicated in red font, and the corresponding aspartate residues in Sp1 or Sp4 are included in “blue boxes.” Note that both D304 and D307 (red box) in Sp3 are part of the core element in the same caspase cleavage motif (see text for details).

In this study, we have identified 12 caspase cleavage positions in Sp3 (human), including D17, D19, D180, D273, D275, D293, D304 (or D307), D326, D344, D530, D543, and D565 (Fig. 11A). By aligning Sp3 amino acid sequences of human, mouse, sheep, and chicken, we found that the critical aspartate residues of the caspase cleavage sites identified in human Sp3 are highly conserved among different species (see Fig. S3 in the supplemental material). During the course of the study, we additionally found that the rates of cleavage of specific sites in Sp3 may vary depending on the particular context. Specifically, we noticed that mutation at the cleavage position D530 in Sp3 affects not only the generation of CP-1 but also the production efficiency of CP-2 (cleavage at D543) and CP-3 (cleavage at D565) (Fig. 6B). These findings imply that the caspase cleavage residues D543 and D565 in the context of a full-length Sp3 seemed to be cleaved inefficiently. For efficient generation of CP-2 or CP-3 by caspases, the full-length Sp3 may need to undergo two sequential cleavages, with the first cleavage at D530 and then the second cleavage at D543 or D565.

Due to the observation that the identified fragments of Sp3 generated by caspase contain the residues S, G, V, T, or L at the N terminus, which correspond to “stabilizing residues” in the N-end rule pathway (57), these caspase-induced fragments of Sp3 could be quite stable in cells (Fig. 1 to 3). Accumulation of cleaved Sp3 fragments in stressed cells may potentially exert some influence on cellular processes. Theoretically, during the apoptotic progression, sequential activation of caspases may cause the generation of diverse repertoire of Sp3 fragments at the early and late stages. These partial cleavage intermediates or complete cleavage products of Sp3 accumulated in cells may profoundly produce different effects on cells. Especially, the IM-1, IM-2, or IM-3 fragment could have biological activities similar to those of the two short Sp3 isoforms, Sp3(aa 286–781) and Sp3(aa 303–781) (Fig. 11A). Further processing of the IM-1, IM-2, or IM-3 fragment by caspases may lead to the generation of two major subfragments, including both the DNA-binding domain and the second activation domain (domain B) (Fig. 11A). These two major subfragments, such as the Sp3(aa 305–530) and Sp3(aa 531–781) fragments generated from IM-1, might have dominant negative effects on the transactivation function of the full-length Sp3, through competing DNA-binding targets or the interaction partners. Conversely, due to ubiquitous expression of Sp3 in cells and the presence of up to 12 caspase cleavage sites in Sp3, it is also possible that these caspase cleavage motifs in Sp3 may function as decoy substrates for active caspases to inhibit or delay the progression of apoptosis. This thinking is reminiscent of the KSHV latency-associated nuclear antigen (LANA) acting as a decoy substrate for caspases to inhibit apoptosis (58). Currently, the precise role of the caspase-mediated cleavage of Sp3 in apoptosis or other cellular processes still remains to be determined.

Since Sp3 shares a high degree of amino acid sequence homology with Sp1 and Sp4, we compared the caspase cleavage sites identified in Sp3 with the corresponding motifs in Sp1 or Sp4. Sequence analysis reveals that 8 out of 12 cleavage positions, including D17, D19, D273, D275, D304 (or 307), D326, D344, and D530, could not be found at the structurally equivalent positions in Sp1 and Sp4 (Fig. 11B). However, three cleavage positions in Sp3, including D180, D543, and D565, could be observed in the corresponding sites of Sp1, including D183, D560, and D577. Only one cleavage position D293 in Sp3 could be found in the corresponding site (D291) of Sp4 (Fig. 11B). To date, there is no report on the proteolytic cleavage of Sp4 by caspases. In the case of Sp1, only a caspase cleavage site located at D183 has been reported previously (59), which corresponds to the caspase cleavage site at D180 in Sp3 (Fig. 11B). Although most of the caspase cleavage sites identified in Sp3 could not be found in the corresponding regions in Sp1 or Sp4, it is still possible that Sp1 or Sp4 may use different sets of caspase cleavage sites to generate functional fragments. Thus, mapping the positions of the caspase cleavage sites in Sp1 and Sp4 would be important for further insights into the structure/function relationship for Sp family proteins.

In summary, we show that the proteolytic cleavage of Sp3 would be a general event during apoptosis (or during the KSHV lytic cycle) and identify at least 12 caspase cleavage sites in Sp3. Further research is needed to elucidate the potential biological activities of cleaved Sp3 fragments in cells.

## MATERIALS AND METHODS

### Cell cultures and reagents.

HH-B2 (14), BCBL1 (7), and BC3 (60) cells were grown in RPMI 1640 medium (no. 11875085; Thermo Fisher Scientific) supplemented with 15% fetal bovine serum (FBS). HH-B2(Tet-On-F-ORF50) is a stable HH-B2 cell clone with a doxycycline-regulated ORF50 gene (61). For viral lytic induction, HH-B2 and BC3 cells were treated with 3 mM SB (no. B5887; Sigma-Aldrich), BCBL1 cells were treated with a combination of 3 mM SB and 30 ng/mL TPA (no. P8139; Sigma-Aldrich), and HH-B2(Tet-On-F-ORF50) cells were treated with 1 μg/mL doxycycline (no. D1822; Sigma-Aldrich). The T lymphocyte cell line Jurkat (62) was cultured in RPMI 1640 medium supplemented with 10% FBS. The human embryonic kidney cell line 293T (63) was cultured in Dulbecco’s modified Eagle medium (no. 11965084; Thermo Fisher Scientific) supplemented with 10% FBS. HKB5/B5 (17), a cell clone derived from 293T cells, was grown in RPMI 1640 medium supplemented with 8% FBS. Staurosporine (no. S4400), camptothecin (no. C9911), and dihydrotanshinone I (no. D0947) were purchased from Sigma-Aldrich (St. Louis, MO, USA). Z-DEVD-FMK (no. FMK004) and Z-VAD (OMe)-FMK (no. tcsc3153) were purchased from R&D Systems (Indianapolis, IN, USA) and Taiclone (Taipei, Taiwan), respectively.

### DNA transfection.

HH-B2 cells were transfected by electroporation using the Neon Transfection System device (no. MPK5000; Thermo Fisher Scientific). Briefly, 4 × 10^6^ cells were mixed with 10 μg of plasmid DNA in 100 μL of buffer B supplied in the Neon Transfection System 100 μL kit (no. MPK10096; Thermo Fisher Scientific) and then electroporated using the setting of 1,400 V/20 ms/2 pulses. DNA transfection in 293T or HKB5/B5 cells was carried out using Lipofectamine 2000 according to the manufacturer’s instruction (no. 11668019; Thermo Fisher Scientific).

### Plasmid construction.

The expression plasmid pCMV-Sp3-FLAG was purchased from GenScript (no. OHu28260D; NM_003111.4; Homo sapiens Sp3, transcript variant 1, mRNA), and pCMV-GFP-Sp3 was constructed by inserting the full-length Sp3 cDNA into pEGFP-C2 (no. 6083-1; Clontech). Point mutations in pCMV-Sp3-FLAG or pCMV-GFP-Sp3 were created by using the QuikChange site-directed mutagenesis kit (no. 200518; Agilent Technologies). To construct the plasmids encoding F-Sp3 or deletion mutants, the corresponding Sp3 DNA fragments were amplified by PCR and then inserted into pFLAG-CMV-2 (no. E7398; Sigma-Aldrich). The plasmids including pCMV-FLAG-ORF50, pCMV-GFP-ORF50(1–590), pCMV-GFP-ORF50(1–490), pE4-luc, ORF56p(−97/−44), ORF21p(−194/−154), ORF60(−71/−32), and IL-10(−141/−102) have been described previously (23, 64, 65).

### Western blot analysis.

Western blotting was performed as described previously (66). Briefly, cell protein extracts were mixed with 3× sodium dodecyl sulfate (SDS) gel loading buffer and boiled for 5 min. Protein samples (20 to 30 μg/well) were loaded and separated in 8 to 10% polyacrylamide gel, and the PageRuler prestained protein ladder (no. 26616; Thermo Fisher Scientific) was used for size standards (10 to 180 kDa) in protein electrophoresis. After electrophoresis, the separated proteins were transferred onto a polyvinylidene difluoride (PVDF) membrane (no. IEVH85R; Millipore), and the membrane was blocked in 5% nonfat milk and incubated with diluted primary antibodies for 2 h at room temperature or 4°C overnight. Antibodies to FLAG (no. SI-A8592; Sigma-Aldrich), K8α (no. sc-57889; Santa Cruz), ORF45 (no. sc-53883; Santa Cruz), Sp3 (no. sc-365220; Santa Cruz), IL-10 (no. bs-0698R; Bioss), actin (no. sc-47778; Santa Cruz), caspase-3 (no. 9662S; Cell Signaling), cleaved caspase-3 (no. 9664S; Cell Signaling), PARP (no. 9542S; Cell Signaling), and GFP (no. G1544; Sigma-Aldrich) were obtained commercially. The anti-ORF50 antibody was generated in our laboratory (61). Membranes were then probed with suitable secondary antibodies, including anti-rabbit IgG antibody conjugated with horseradish peroxidase (no. AP132P; Sigma-Aldrich) and anti-mouse IgG antibody conjugated with horseradish peroxidase (no. AP124P; Sigma-Aldrich). After extensive washing, the Western Lightning chemiluminescence reagent (no. NEL105001EA: PerkinElmer) was used for detecting the antigen-antibody complex on membranes.

### Luciferase assays.

HKB5/B5 cells were seeded on 24-well plates (7 × 10^5^ cells/well) 1 day before transfection. Each transfection used a fixed amount (0.6 μg) of plasmid DNA including effectors and reporter constructs. At 45 h posttransfection, transfected cells were harvested and luciferase reporter activities were measured using the luciferase reporter assay system (no. E1501; Promega). All of the experiments were performed in duplicate and repeated at least three times. Fold activation of the promoter activity was calculated as luciferase activity in the presence of effectors divided by those in the presence of the empty vector. Data were presented as mean ± standard deviation (SD). The synergistic index was defined as mentioned previously (67) and was calculated as the ratio of the luciferase activity of the reporter construct in the presence of both ORF50 and specific F-Sp3 mutant over the sum of the activity of the reporter construct alone and the increases in this activity caused by the expression of ORF50 and specific F-Sp3 mutant individually. Student’s *t* test was used to evaluate the significance of differences between samples. A *P* value of less than 0.05 was considered statistically significant.

### Electrophoretic mobility shift assay.

Total protein extracts of 293T cells transfected with the expression plasmids were prepared as described previously (68). Annealed double-stranded DNA oligonucleotides were end-labeled with biotin-11-UTP using terminal deoxynucleotidyl transferase (no. 89818; Pierce). Binding reactions contained 12 μg of total protein extracts in a solution containing 10 mM HEPES (pH 7.5), 50 mM NaCl, 2 mM MgCl_2_, 2.5 μM ZnSO_4_, 0.5 mM EDTA, 1 mM dithiothreitol, 15% glycerol, and 0.3 μg poly(dIdC) in a total volume of 20 μl. Antibodies to FLAG (no. SI-A2220; Sigma-Aldrich), GFP (no. G1544; Sigma-Aldrich), and actin (no. sc-47778; Santa Cruz) were used for supershift studies.

### Coimmunoprecipitation.

Coimmunoprecipitation experiments were performed as mentioned previously (23). Briefly, the plasmids including pEGFP-C2, pCMV-GFP-ORF50(1-590), or pCMV-GFP-ORF50(1-490) were cotransfected with the F-Sp3(533-781) construct into 293T cells for 24 h. Cell lysates were prepared in the immunoprecipitation assay buffer (50 mM Tris-Cl [pH 7.6], 150 mM NaCl, 1 mM EDTA, 1% Triton X-100, and 5 μl/mL protease inhibitor cocktail), and then mixed with GFP-Trap MA beads (no. gtma-100; ChromoTek). After immunoprecipitation, the resultant immunoprecipitates were analyzed by Western blotting.
