# Supplementary material for: Caspase-Mediated Cleavage of the Transcription Factor Sp3: Possible Relevance to Cancer and the Lytic Cycle of Kaposi’s Sarcoma-Associated Herpesvirus
Source: Microbiol Spectr. 2022 Jan 12;10(1):e01464-21. doi: 10.1128/spectrum.01464-21 (PMC8754129; doi:10.1128/spectrum.01464-21)
Supplement: SUPPLEMENTAL FILE 1 — Supplemental material. Download SPECTRUM01464-21_Supp_1_seq14.pdf, PDF file, 3.7 MB [file spectrum01464-21_supp_1_seq14.pdf]

SUPPLEMENTAL INFORMATION

Fig. 2

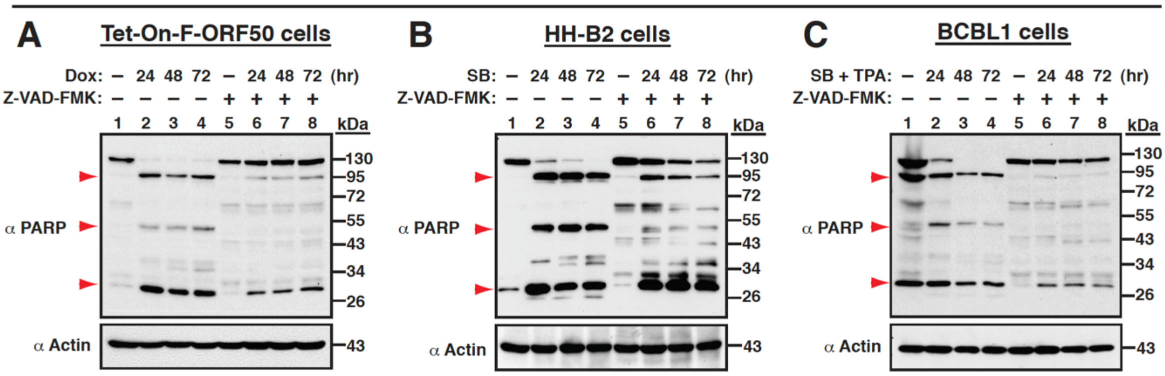

Fig. 3

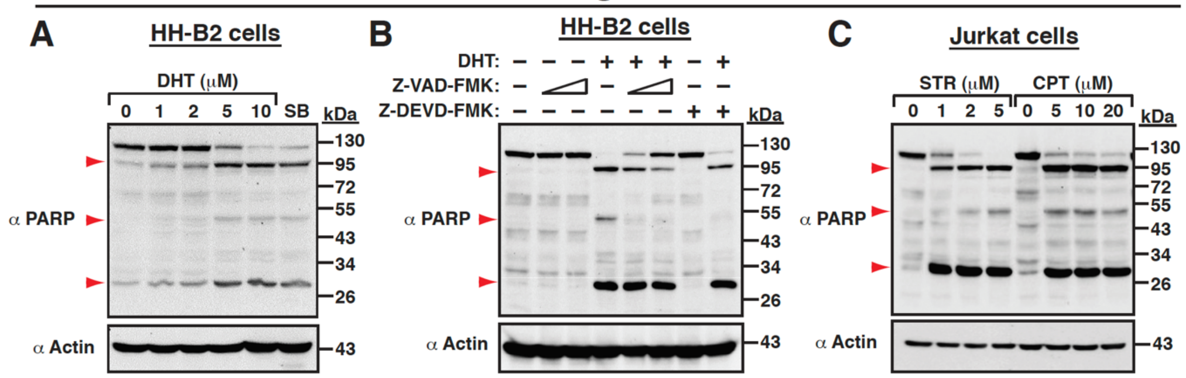

Fig. 4A

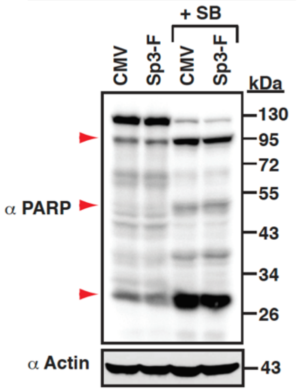

Supplementary Fig. S1. Full scan (uncropped) images of Western blotting of PARP presented in Fig. 2, 3 and 4A. Western blot analysis using the anti-PARP antibody (#9542S; Cell Signaling) revealed that at least three cleaved PARP fragments (89, 50 and 28 kDa; red arrowheads) could be detected in cells undergoing apoptosis.

A. Predicted cleavage sites for caspase-1 in Sp3

rank	position	site	N fragment	C fragment	frequency score	similarity maxscore	similarity maxsite	average score	specificity
1	540 to 545	ENAD.SP	55.4 kD	26.5 kD	0.250	54.545	HLADSP	13.636	>99%
2	527 to 532	NSID.SA	54.1 kD	27.8 kD	0.250	42.424	DEIDHA	10.606	>99%
3	16 to 21	LDVD.SG	2.1 kD	79.8 kD	0.083	60.000	LVVDNG	5.000	>99%
4	553 to 558	EEPD.PE	56.9 kD	25.0 kD	0.054	56.250	EEEDGE	3.013	>99%
5	562 to 567	LSGD.ST	57.9 kD	24.0 kD	0.033	58.621	LSSDFT	1.954	>99%
6	177 to 182	QSAD.GQ	17.7 kD	64.2 kD	0.025	48.485	ELPDGQ	1.212	>99%
7	364 to 369	HSSD.LQ	36.8 kD	45.1 kD	0.027	42.424	YQSDNQ	1.136	>99%
8	304 to 309	DSSD.NS	30.4 kD	51.5 kD	0.021	45.161	DETDSK	0.968	>99%
9	341 to 346	VTID.ST	34.4 kD	47.5 kD	0.008	35.714	IEADSE	0.298	>95%
10	579 to 584	QVVD.EE	59.8 kD	22.1 kD	0.007	33.333	LVVDNG	0.248	>95%
11	290 to 295	INAD.GH	29.0 kD	52.9 kD	0.004	50.000	IEADSE	0.208	>95%
12	50 to 55	AAQD.TQ	5.1 kD	76.8 kD	0.004	45.161	NMQDSQ	0.202	>95%
13	190 to 195	GSSD.NG	19.0 kD	62.9 kD	0.005	36.667	AAVDGG	0.196	>95%
14	721 to 726	AARD.DT	76.1 kD	5.8 kD	0.003	37.500	AQRDSH	0.112	>95%
15	93 to 98	ATGD.LA	9.0 kD	72.9 kD	0.002	39.286	ALDDLI	0.088	>95%
16	722 to 727	ARDD.TL	76.2 kD	5.7 kD	0.001	53.571	ALDDLI	0.080	>95%
17	583 to 588	EEGD.QQ	60.3 kD	21.6 kD	0.002	43.750	EEEDGE	0.070	>95%
18	691 to 696	MRSD.HL	72.9 kD	9.0 kD	0.001	43.750	LESDYF	0.039	>95%
19	270 to 275	NSVD.LD	27.1 kD	54.8 kD	0.001	22.581	NMQDSQ	0.034	>95%
20	663 to 668	TRSD.EL	69.4 kD	12.5 kD	0.000	34.615	ALADSL	0.015	>95%

B. Predicted cleavage sites for caspase-3 in Sp3

rank	position	site	N fragment	C fragment	frequency score	similarity maxscore	similarity maxsite	average score	specificity
1	16 to 21	LDVD.SG	2.1 kD	79.8 kD	0.924	79.310	VEVDSG	73.255	>99.9%
2	304 to 309	DSSD.NS	30.4 kD	51.5 kD	0.835	72.414	DSSDSE	60.434	>99.9%
3	272 to 277	VDLD.SL	27.3 kD	54.6 kD	0.693	78.571	LDVDSL	54.420	>99%
4	527 to 532	NSID.SA	54.1 kD	27.8 kD	0.297	84.615	SSVDSA	25.159	>99%
5	553 to 558	EEPD.PE	56.9 kD	25.0 kD	0.097	67.742	EEPDSA	6.591	>99%
6	341 to 346	VTID.ST	34.4 kD	47.5 kD	0.074	63.333	DTLDST	4.702	>99%
7	540 to 545	ENAD.SP	55.4 kD	26.5 kD	0.054	83.871	EEADSP	4.508	>99%
8	177 to 182	QSAD.GQ	17.7 kD	64.2 kD	0.050	76.667	ESVDGQ	3.811	>99%
9	270 to 275	NSVD.LD	27.1 kD	54.8 kD	0.041	60.714	DSVDLA	2.484	>95%
10	562 to 567	LSGD.ST	57.9 kD	24.0 kD	0.039	58.621	LSSDFT	2.305	>95%
11	325 to 330	TDTD.LF	32.7 kD	49.2 kD	0.031	74.194	TETDAF	2.277	>95%
12	190 to 195	GSSD.NG	19.0 kD	62.9 kD	0.042	46.667	ESMDSG	1.965	>95%
13	301 to 306	QAMD.SS	30.1 kD	51.8 kD	0.024	71.429	DALDSS	1.741	>95%
14	156 to 161	PGSD.SS	15.5 kD	66.4 kD	0.021	55.556	SESDSS	1.192	>95%
15	316 to 321	VSPD.IN	31.7 kD	50.2 kD	0.018	55.172	IVPDIA	1.014	>95%
16	77 to 82	DEEE.AA	7.8 kD	74.1 kD	0.014	64.516	DEDDSA	0.932	>95%
17	583 to 588	EEGD.QQ	60.3 kD	21.6 kD	0.016	55.172	EEIDAQ	0.894	>95%
18	14 to 19	AALD.VD	1.9 kD	80.0 kD	0.011	60.000	SALDGD	0.668	>95%
19	93 to 98	ATGD.LA	9.0 kD	72.9 kD	0.010	48.276	STTDLT	0.471	>95%
20	579 to 584	QVVD.EE	59.8 kD	22.1 kD	0.008	51.724	DLVDAE	0.404	>95%

C. Predicted cleavage sites for caspase-7 in Sp3

rank	position	site	N fragment	C fragment	frequency score	similarity maxscore	similarity maxsite	average score	specificity
1	272 to 277	VDLD.SL	27.3 kD	54.6 kD	0.161	51.724	DELDSI	8.341	>99%
2	304 to 309	DSSD.NS	30.4 kD	51.5 kD	0.062	60.000	DQTDSS	3.735	>99%
3	16 to 21	LDVD.SG	2.1 kD	79.8 kD	0.046	53.571	MEVDAA	2.474	>99%
4	341 to 346	VTID.ST	34.4 kD	47.5 kD	0.035	40.000	VEVDAP	1.407	>99%
5	553 to 558	EEPD.PE	56.9 kD	25.0 kD	0.025	51.429	DEPDSP	1.282	>99%
6	316 to 321	VSPD.IN	31.7 kD	50.2 kD	0.027	45.946	FDPDIH	1.243	>99%
7	540 to 545	ENAD.SP	55.4 kD	26.5 kD	0.013	53.125	DMVDSP	0.709	>99%
8	527 to 532	NSID.SA	54.1 kD	27.8 kD	0.008	60.714	SSLDGA	0.513	>99%
9	93 to 98	ATGD.LA	9.0 kD	72.9 kD	0.008	48.276	STTDLT	0.366	>95%
10	325 to 330	TDTD.LF	32.7 kD	49.2 kD	0.007	46.667	DETDLA	0.332	>95%
11	156 to 161	PGSD.SS	15.5 kD	66.4 kD	0.006	40.625	PELDGS	0.238	>95%
12	364 to 369	HSSD.LQ	36.8 kD	45.1 kD	0.005	46.667	DSEDLK	0.221	>95%
13	275 to 280	DSLG.LS	27.5 kD	54.4 kD	0.003	46.667	DSVDFS	0.127	>95%
14	63 to 68	AATC.SK	6.4 kD	75.5 kD	0.004	28.125	DATDGK	0.121	>95%
15	177 to 182	QSAD.GQ	17.7 kD	64.2 kD	0.002	76.667	ESVDGQ	0.117	>95%
16	77 to 82	DEEE.AA	7.8 kD	74.1 kD	0.002	53.125	DQPDAA	0.115	>95%
17	190 to 195	GSSD.NG	19.0 kD	62.9 kD	0.002	45.161	GSSDPL	0.110	>95%
18	379 to 384	EETQ.AQ	38.4 kD	43.5 kD	0.002	45.161	AETDGQ	0.104	>95%
19	562 to 567	LSGD.ST	57.9 kD	24.0 kD	0.003	35.484	DEGDSL	0.094	>95%
20	366 to 371	SDLQ.GN	37.0 kD	44.9 kD	0.001	61.290	SEVDGN	0.070	>95%

D. Predicted cleavage sites for caspase-8 in Sp3

rank	position	site	N fragment	C fragment	frequency score	similarity maxscore	similarity maxsite	average score	specificity
1	16 to 21	LDVD.SG	2.1 kD	79.8 kD	2.671	66.667	IETDSG	178.063	>99.9%
2	325 to 330	TDTD.LF	32.7 kD	49.2 kD	0.128	46.667	DETDLA	5.983	>99%
3	540 to 545	ENAD.SP	55.4 kD	26.5 kD	0.112	53.125	DMVDSP	5.960	>99%
4	553 to 558	EEPD.PE	56.9 kD	25.0 kD	0.100	56.250	EEEDGE	5.609	>99%
5	562 to 567	LSGD.ST	57.9 kD	24.0 kD	0.142	39.286	IEADSE	5.596	>99%
6	304 to 309	DSSD.NS	30.4 kD	51.5 kD	0.111	43.333	DSIDSF	4.815	>99%
7	272 to 277	VDLD.SL	27.3 kD	54.6 kD	0.080	42.857	IDVDVS	3.434	>99%
8	14 to 19	AALD.VD	1.9 kD	80.0 kD	0.036	41.935	AEVDGD	1.493	>99%
9	93 to 98	ATGD.LA	9.0 kD	72.9 kD	0.021	41.935	DMGDLV	0.896	>99%
10	190 to 195	GSSD.NG	19.0 kD	62.9 kD	0.014	46.667	ESMDSG	0.665	>95%
11	290 to 295	INAD.GH	29.0 kD	52.9 kD	0.012	50.000	IEADSE	0.594	>95%
12	527 to 532	NSID.SA	54.1 kD	27.8 kD	0.009	60.714	SSLDGA	0.519	>95%
13	341 to 346	VTID.ST	34.4 kD	47.5 kD	0.011	37.931	VETDSE	0.405	>95%
14	270 to 275	NSVD.LD	27.1 kD	54.8 kD	0.009	34.483	DLVDAE	0.295	>95%
15	177 to 182	QSAD.GQ	17.7 kD	64.2 kD	0.004	53.125	REMDGQ	0.202	>95%
16	301 to 306	QAMD.SS	30.1 kD	51.8 kD	0.003	60.000	ESMDSG	0.192	>95%
17	583 to 588	EEGD.QQ	60.3 kD	21.6 kD	0.004	43.750	EEEDGE	0.175	>95%
18	323 to 328	TNTD.TD	32.5 kD	49.4 kD	0.002	50.000	TLTDSS	0.107	>95%
19	118 to 123	TIKD.EA	11.6 kD	70.3 kD	0.001	46.667	TISDSP	0.053	>95%
20	364 to 369	HSSD.LQ	36.8 kD	45.1 kD	0.002	30.000	DETDLA	0.051	>95%

Supplementary Fig. S2. The predicted probability of the cleavage sites for caspase-1 (A), caspase-3 (B), caspase-7 (C) and capase-8 (D) in Sp3 using a web-based prediction tool (SitePrediction; https://www.dmbr.ugent.be/prx/bioit2-public/SitePrediction/).

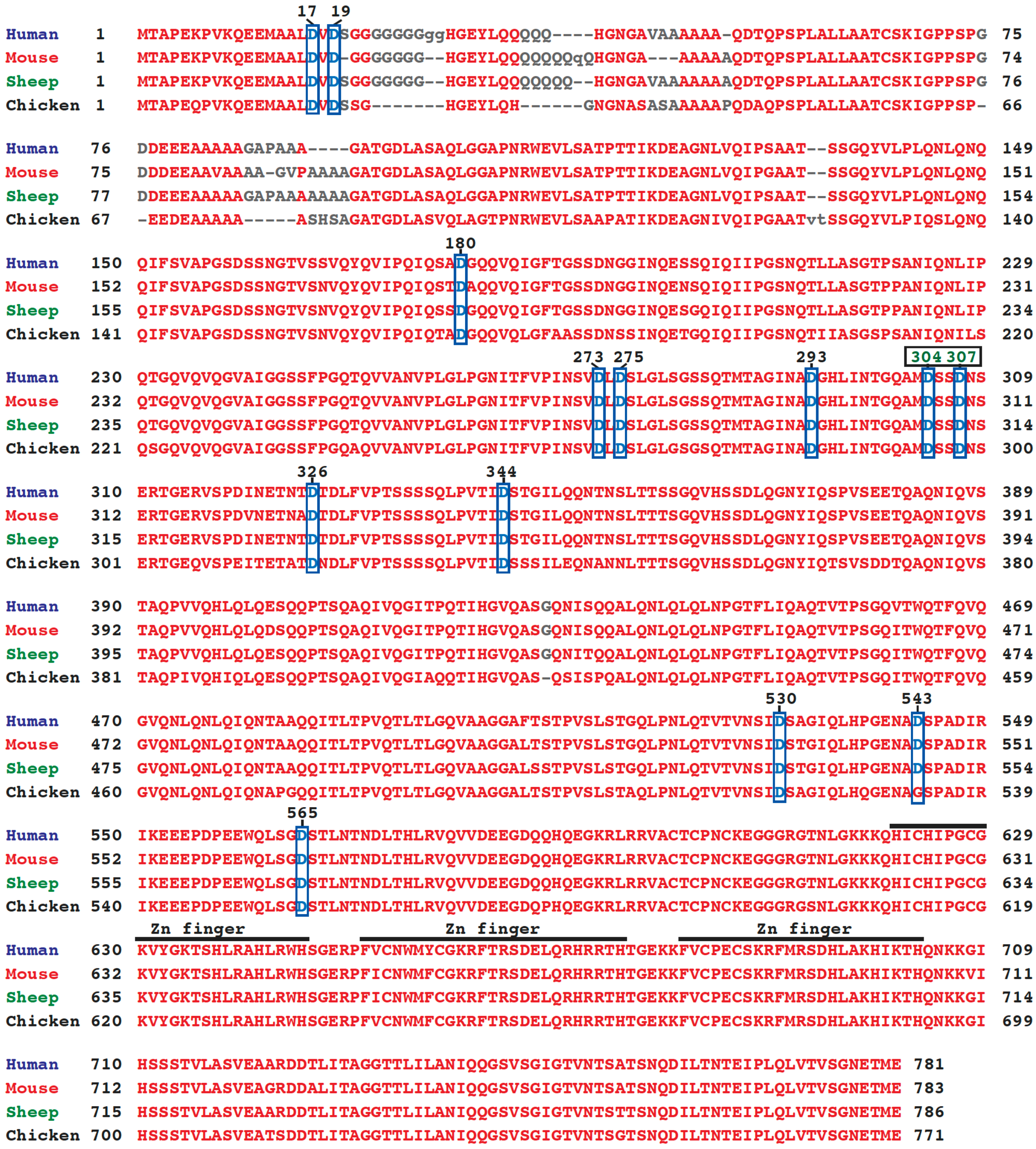

Supplementary Fig. S3. Conservation of the caspase cleavage sites in Sp3 proteins from human (RefSeq: NP_003102.1), mouse (RefSeq: NP_001018052.1), sheep (RefSeq: XP_027820637.1) and chicken (RefSeq: NP_989934.1). The protein sequence alignment of these Sp3 proteins were drawn using a alignment tool (COBALT; https://www.ncbi.nlm.nih.gov/tools/cobalt/re_cobalt.cgi). The critical aspartate (D) residues of the caspase cleavage sites in Sp3 proteins are indicated in blue font, and numbers above these aspartate residues represent the amino acid positions of human Sp3. The amino acid residues at D304 and D307 (green color) are essential for making up a functional caspase cleavage motif (See text for details).
